# Antibiotic use in Kenyan public hospitals: Prevalence, appropriateness and link to guideline availability

**DOI:** 10.1016/j.ijid.2020.07.084

**Published:** 2020-10

**Authors:** Michuki Maina, Paul Mwaniki, Edwin Odira, Nduku Kiko, Jacob McKnight, Constance Schultsz, Mike English, Olga Tosas-Auguet

**Affiliations:** a KEMRI-Wellcome Trust Research Programme, Health Services Research Group, PO Box 43640-00100 Nairobi, Kenya; b Department of Global Health, Amsterdam UMC, University of Amsterdam, Postbus 22660,1100 DD Amsterdam, The Netherlands; c Department of Surgery, Kitui County Referral Hospital PO Box 33-90200 Kitui, Kenya; d Department of Medicine, Kenyatta National Hospital, PO Box 20723-00202 Nairobi, Kenya; e The University of Oxford, Nuffield Department of Medicine, Peter Medawar Building for Pathogen Research, South Parks Road, OX1 3SY, Oxford, United Kingdom; f Amsterdam Institute for Global Health and Development- AHTC, Tower C4, Paasheuvelweg 25 1105 BP Amsterdam, The Netherlands

**Keywords:** Antimicrobial resistance, Antibiotics, Point prevalence survey

## Abstract

**Objective:**

To examine prescription patterns and explore to what extent guidelines are available and how they might influence treatment appropriateness among hospitalised patients in Kenyan hospitals.

**Methods:**

Data on antimicrobial usage were collected from hospitalised patients across 14 Kenyan public hospitals. For each prescription, appropriateness of treatment was defined using available local and international treatment guidelines and through consensus with local medical specialists. Association between appropriate treatment, guideline availability and other possible explanatory factors was explored using univariate and multiple regression analysis.

**Results:**

There were 1675 (46.7%) of the 3590 hospitalised patients on antimicrobials with 3145(94%) of the 3363 antimicrobial prescriptions being antibiotics. Two patients (0.1%), had treatment based on available antibiotic susceptibility tests. Appropriate treatment was assessed in 1502 patients who had a single diagnosis. Of these, 805 (53.6%) received appropriate treatment. Physical availability of treatment guidelines increased the odds of receiving appropriate treatment Odds Ratio 6.44[95% CI 4.81–8.64].

**Conclusion:**

Appropriate antibiotic prescription remains a challenge in Kenyan public hospitals. This may be improved by the availability of context-specific, up-to-date, and readily accessible treatment guidelines across all the departments, and by providing better diagnostic support.

## Highlights

• We report findings from a point prevalence survey across 14 Kenyan public hospitals.
• About half of the hospitalised patients received appropriate antibiotic therapy.
• Laboratory investigations supported less than 1% of the antibiotic prescriptions.
• Physical availability of treatment guidelines influenced treatment appropriateness.
• There is need for context-specific, up-to-date, and accessible treatment guidelines.

## Background

Antimicrobial resistance (AMR) is an emerging global challenge that is thought to account for more than 700,000 deaths annually (O’neill, 2014). Certain practices that are common in resource-limited settings can fuel resistance. Inappropriate use of antibiotics is highly problematic, especially where they are availed over the counter without prescriptions. Similarly, the availability of falsified or substandard drugs is a significant issue (O’neill, 2014, Ayukekbong et al., 2017). In hospital settings, inappropriate use has been fuelled by lack of surveillance and diagnostic capabilities, poor antibiotic stewardship activities and lack of treatment guidelines (Petti et al., 2006, Elbireer et al., 2013, Doron and Davidson, 2011, Paterson, 2006).

Efforts to improve the appropriateness of antibiotic use rely on the availability of relevant usage data. Unfortunately, there is a lack of data on antimicrobial use in African countries compared to other regions (Ahoyo et al., 2014, Versporten et al., 2018). To measure antibiotic use and quality of prescriptions at hospital and patient level, point prevalence surveys (PPS) have been extensively used (Gharbi et al., 2016, Okoth et al., 2018). They are easy to administer and, in addition to generating data on antibiotic use, can highlight other problematic aspects of quality of care, including the quality of prescription. They also offer a useful tool for audit and feedback on antibiotic use in hospitals to improve decision making and strengthen antibiotic stewardship activities.

The Government of Kenya in 2017 launched the national plan on prevention and containment of antimicrobial resistance. One of the strategies is to optimise the use of antimicrobials. This can be achieved through increasing compliance to reporting of antimicrobial consumption across the country. (Republic of Kenya, 2017)

Some studies in Kenya indicate a high prevalence of antibiotic use in both inpatient and outpatient settings of more than 80%. Also highlighted in these studies are challenges with the quality of prescriptions, including the use of proprietary drug names and incomplete prescriptions (Mulwa et al., 2015, Okoth et al., 2018). These studies were, however, from single hospitals. We set out to conduct an antibiotic point prevalence survey across 14 public hospitals in Kenya, examine prescription quality and explore to what extent guidelines are available and how they might influence treatment appropriateness.

## Methods

This study took as its starting point the methods developed for the Global Point Prevalence Survey of Antimicrobial Consumption and Resistance (GLOBAL-PPS), which has been widely used and for which tools are freely available. (Versporten et al., 2018, GLOBAL-PPS, 2015).

### Setting

The point prevalence survey was carried out between February and April 2018 across 14 public hospitals in Kenya with a total bed capacity of 4152 (Maina et al., 2019).These hospitals are distributed across the central, eastern and western parts of the country in high and low malaria prevalence regions. HIV prevalence ranges between 2 and 16% across the counties where these hospitals are located. The bed capacity, number of specialists, catchment population and the HIV prevalence, are provided as a supplement. (Supplement 1).These hospitals provide multi-speciality care and were selected purposively as part of a collaboration between the Ministry of Health and the KEMRI Wellcome Trust Research Programme (English et al., 2017a). During the surveys, the months of March to May 2018, which are the long rains period in Kenya experienced heavy rainfall and flooding in most parts of the country (Ministry of Environment and Forestry, 2018). This rainy period sees increased hospitalisations due to malaria and diarrhoeal diseases in Kenya (Apat et al., 2017, Tornheim et al., 2010).

### Data collection

#### Ward-level

At ward level, data were collected on the department type, bed and patient numbers. These data were used to compute bed occupancy. As a modification to the global PPS, wards were grouped into five departments, namely adult medical, adult surgical, paediatric medical, paediatric surgical or neonatal. In facilities with more than one ward in these groupings, all were surveyed, and results pooled at group level. The data collection tool is provided as a supplement (Supplement 2)

#### Patient-level

At the patient level, data were collected on the patients' age, sex, date of admission and diagnoses. For the diagnosis, a total of 46 possible options were provided, 45 of these were categorised by the anatomical system involved. The last category was when the prescription was not supported by documentation of any diagnosis for which antibiotics are indicated. This category was labelled as “Conditions for which antibiotics are not indicated”. Data were also collected on the antimicrobial type, dose and duration of treatment. Microbiology, antibiotic susceptibility and biomarker (C-reactive protein; procalcitonin) test results used to inform the diagnosis and treatment, were also documented. Supplement 3 provides the patient-level data collection tool and the list of diagnoses.

### Data collection process

Data were collected by clinicians trained on the study methodology. There were three data collection teams, each with 5–6 members under the leadership of a clinical pharmacist. The survey followed standard operating procedures and data capture forms currently in use as part of the Global PPS with some context-appropriate modifications. One modification included data collection on all weekdays mainly due to logistical reasons. Data were not collected on weekends and holidays. The survey procedures are given in Supplement 4.

### Inclusion and exclusion criteria

#### Ward-level

All wards from paediatric (medical and surgical), adult (medical and surgical including obstetrics and gynaecology) and neonatal were included in the study. Psychiatric, Ear Nose and Throat (ENT), renal, neurosurgery, eye and intensive care units, were not present in all hospitals and were excluded. One large hospital included in the survey only provides maternal and neonatal care. Data from this facility were included in relevant analyses.

#### Patient-level

Data were collected from all hospitalised patients on antimicrobial treatment or prophylaxis, even if the drug was not administered on the day of the survey. Outpatients and day case admissions were excluded.

### Electronic data entry

At each ward, de-identified data were entered from paper medical records into REDCap using laptop computers. REDCap® (Research Electronic Data Capture) is a secure, web-based application that supports data capture (Harris et al., 2009). In-built range, error and validity checks were employed at the point of data entry.

### Data analysis

At ward level, proportions were computed to establish bed occupancy and the percentage of hospitalised patients on antimicrobial treatment. At the patient level, since each patient was eligible for a maximum of five diagnoses, specific diagnoses were computed as a proportion of the total number of diagnoses.

For analysis of antimicrobial agents, these were grouped into ten main groups based on their Anatomical Therapeutic Chemical Classification System (ATC) (World Health Organization, 2015). In addition to the groups based on the ATC, we generated a new group that was a combination of benzylpenicillin and gentamicin to capture all the prescriptions in which this combination was used. This combination is the expected national first-line treatment for many severe paediatric and neonatal infections in Kenya (World Health Organization, 2013, Ministry of Health, 2016).

#### Antibiotic treatment appropriateness

In this study, treatment appropriateness is defined as any prescription which is in keeping with (i) treatment guidelines, (ii) consensus of local expert opinion or (iii) bacterial speciation and antibiotic susceptibility tests.

To determine treatment appropriateness, we first searched for all available Kenyan (hospital-based or national) guidelines for each of the 45 diagnostic classifications in the Global-PPS. For the conditions without local guidance, we searched for guidelines from the World Health Organisation, the Infectious Diseases Society of America, the National Institute for Health and Care Excellence and guidelines from international medical associations, e.g. The European Society of Intensive Care Medicine. We chose the international guidelines through consensus with local medical specialists since these guidelines are commonly used in Kenya for teaching. We documented which of these local/international guidelines were physically available in the relevant wards during the survey.

De-identified individual patient data including diagnoses and antibiotic prescription were then shared with a speciality consultant relevant to the ward they were admitted to (either a surgeon, physician, obstetrician or a paediatrician). None of the consultants worked in the participant hospitals, and all were blinded as to the hospital source of the data. Based on the reference guidelines, these consultants reviewed the individual prescriptions and classified treatment as inappropriate if it was not in keeping with the guidelines, or if it involved redundant antibiotic combinations (where antibiotics have a similar spectrum of cover). The principal investigator (PI), identified a sample of ten medical records from each of the departments and independently assessed appropriateness using the same approach. This independent, duplicate sample was used to check for congruency with the decisions made by the specialists. In cases where the decision by the PI and that of the specialists were not in agreement and consensus was not forthcoming, the opinion of an infectious disease specialist was sought.

To establish the appropriateness by disease condition, a subset of the data was used. The data were for those patients with only a single diagnosis that warranted antibiotic treatment. These individuals were used to calculate the proportions of appropriate treatment by disease condition.

#### Factors associated with treatment appropriateness

In keeping with our primary interest, we explored whether the physical availability of treatment guidelines was associated with treatment appropriateness and included the number of comorbidities, gender, and duration of hospital stay (period between admission and day of survey) as additional explanatory factors. Duration of hospital stay and comorbidities were included in following the hypothesis that patients that spend longer in hospital are more likely to have a more significant number of prescriptions, and likely to have more complex illness (Nobili et al., 2011). The complexity of the illness and the number of prescriptions may possibly have an influence on the appropriateness of the prescription choice. Duration of hospital stay was centred and scaled to improve model stability and reduce the problems of model convergence (Pinheiro et al., 1995). Frequency tables and graphs were used to present the data. Statistical analysis was conducted in R (R Core Team, 2013).

## Results

Data are presented for 14 public hospitals in Kenya. Ten of the hospitals had five departments (adult medical, adult surgical, paediatric medical, paediatric surgical and neonatal unit). One hospital (H16) is a maternity hospital, therefore had no paediatric and adult male units. Hospitals H9, H10 and H11 had no paediatric surgical units. There were no adult surgical patients hospitalised in H10 during the survey. A total of 3590 patients were hospitalised during the survey, with a median of 230 patients, interquartile range (IQR[137] across the hospitals. The bed capacity varied across the hospitals with a median of 297 beds IQR[137]. The median occupancy was 87% IQR[39]. Table 1 provides more description.

**Table 1:** Hospital bed occupancy and antimicrobial prescription proportions.

	Adult Medical			Adult Surgical			Neonatal			Paediatric Medical			Paediatric Surgical				Total		
Hospital	Beds Occupancy (%)	Patients No	No on Antimicrobials (%)	Beds Occupancy (%)	Patient No	No on Antimicrobials (%)	Beds Occupancy (%)	Patient No	No on Antimicrobials (%)	Beds Occupancy (%)	Patient No	No on Antimicrobials (%)	Beds Occupancy (%)	Patient No	No on Antimicrobials (%)	Beds Occupancy (%)		Patient No	No on Antimicrobials (%)
H1	96 (53.1)	51	36 (70.6)	69 (108.7)	75	53 (70.7)	8 (212.5)	17	16 (94.1)	16 (131.2)	21	18 (85.7)	15 (73.3)	11	11 (100.0)	204 (85.8)		175	134 (76.6)
H2	233 (69.5)	162	48 (29.6)	147 (81.6)	120	24 (20.0)	109 (38.5)	42	17 (40.5)	70 (52.9)	37	17 (45.9)	24 (62.5)	15	3 (20.0)	583 (64.5)		376	109 (29.0)
H3	118 (50.0)	59	25 (42.4)	102 (89.2)	91	73 (80.2)	14 (207.1)	29	17 (58.6)	48 (37.5)	18	15 (83.3)	18 (61.1)	11	2 (18.2)	300 (69.3)		208	132 (63.5)
H4	72 (70.8)	51	15 (29.4)	120 (57.5)	69	21 (30.4)	58 (43.1)	25	1 (4.0)	27 (63.0)	17	12 (70.6)	24 (20.8)	5	3 (60.0)	301 (55.5)		167	52 (31.1)
H5	89 (101.1)	90	33 (36.7)	93 (78.5)	73	28 (38.4)	76 (67.1)	51	14 (27.5)	23 (121.7)	28	22 (78.6)	13 (115.4)	15	6 (40.0)	294 (87.4)		257	103 (40.1)
H6	184 (129.9)	239	52 (21.8)	95 (102.1)	97	34 (35.1)	45 (106.7)	48	15 (31.2)	50 (132.0)	66	53 (80.3)	39 (38.5)	15	3 (20.0)	413 (112.6)		465	157 (33.8)
H7	69 (88.4)	61	41 (67.2)	34 (111.8)	38	34 (89.5)	7 (228.6)	16	16 (100.0)	29 (72.4)	21	19 (90.5)	6 (100.0)	6	3 (50.0)	145 (97.9)		142	113 (79.6)
H8	99 (105.1)	104	47 (45.2)	39 (133.3)	52	39 (75.0)	31 (145.2)	45	20 (44.4)	33 (63.6)	21	18 (85.7)	26 (34.6)	9	8 (88.9)	228 (101.3)		231	132 (57.1)
H9	181 (67.4)	122	41 (33.6)	143 (86.7)	124	66 (53.2.2)	78 (62.8)	49	18 (36.7)	56 (58.9)	33	32 (97.0)	NA	NA	NA	458 (71.6)		328	157 (47.9)
H10	81 (135.8)	110	47 (42.7)	NA	NA	NA	35 (105.7)	37	21 (56.8)	38 (176.3)	67	56 (83.6)	NA	NA	NA	154 (139.0)		214	124 (57.9)
H11	157 (110.2)	173	73 (42.2)	24 (129.2)	31	31 (100.0)	31 (125.8)	39	24 (61.5)	43 (120.9)	52	33 (63.5)	NA	NA	NA	255 (115.7)		295	161 (54.6)
H13	108 (142.6)	154	51 (33.1)	97 (106.2)	103	35 (34.0)	106 (72.6)	77	11 (14.3)	28 (153.6)	43	30 (69.8)	20 (105.0)	21	4 (19.0)	359 (110.9)		398	131 (32.9)
H14	54 (53.7)	29	24 (82.8)	55 (98.2)	54	49 (90.7)	14 (57.1)	8	7 (87.5)	16 (37.5)	6	6 (100.0)	16 (50.0)	8	5 (62.5)	155 (67.7)		105	91 (86.7)
H16	171 (63.2)	108	5 (4.6)	62 (93.5)	58	41 (70.7)	84 (75.0)	63	33 (52.4)	NA	NA	NA	NA	NA	NA	317 (72.2)		229	79 (34.5)
Total	1712 (88.4)	1513	538 (35.6)	1080 (91.2)	985	528 (53.3)	696 (78.4)	546	230 (42.1)	477 (90.1)	425	331 (77.9)	201 (57.7)	116	48 (41.4)	4166 (86.2)		3590	1675 (46.7)

NA- Indicates this department was absent.

### Overall antimicrobial usage

There were 1675 (47%) of the 3590 hospitalised patients on treatment with at least one antimicrobial agent. Of all the hospital departments, the adult medical departments had the highest number of hospitalised patients (1513). Of these, 538 (35.6%) were on at least one antimicrobial. In the adult surgical, neonatal, paediatric medical and paediatric surgical wards there were 990, 546, 425 and 116 patients respectively with 528 (53.3%), 230 (42.1%), 331 (77.9%) and 48 (41.4%) patients on antibiotics respectively.

### Antimicrobial and antibiotic use

There were 3363 antimicrobial prescriptions (median 2 per patient). Of these, 3145 (94%) were antibiotic prescriptions, with 95 anti-parasitic agents (3%), 80 antivirals (2%) and 43 antifungal agents (1%) accounting for the remainder. This report, therefore, focusses on antibiotic prescriptions as they were the most common.

**Figure 1:**
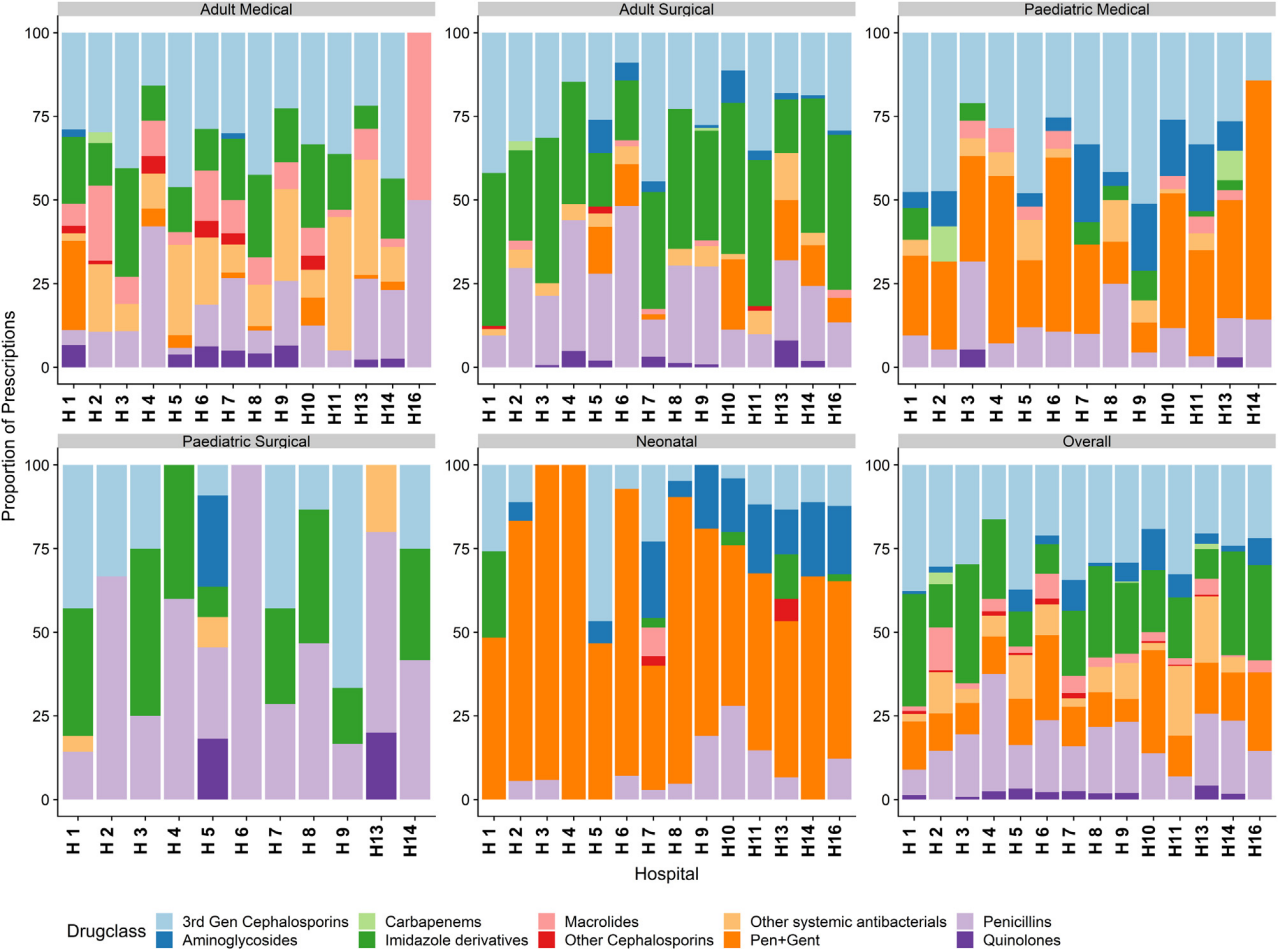
Bar charts denoting the proportions of drug prescriptions by drug ATC class across 14 hospitals by departments and overall. Also included is a combination of benzylpenicillin and gentamicin.

The 3145 antibiotic prescriptions were made for 1675 patients. These included a total of 1078, 816, 305, 450 and 92 antibiotic prescriptions in the adult surgical, adult medical, neonatal, paediatric medical and paediatric surgical units respectively. Of these total prescriptions, 404 (13%) were a combination of benzylpenicillin and gentamicin. Cephalosporins were the most common prescriptions accounting for 772 (26%) of the remaining 2741 prescriptions. They were followed by nitroimidazole derivatives, mainly metronidazole, with 585 (20%) prescriptions. Combinations of benzylpenicillin and gentamicin were predominant in the neonatal unit with 178 (58%) of the 305 prescriptions. The 3rd generation cephalosporins in the adult medical units represented 263/816 (32%) of prescriptions. Two (0.1%) of the 1675 patients in the study (one adult medical and one paediatric medical) had treatment based on antibiotic susceptibility tests. Figure 1 illustrates the proportion of prescriptions by department and hospital.

### Diagnoses warranting treatment

**Figure 2:**
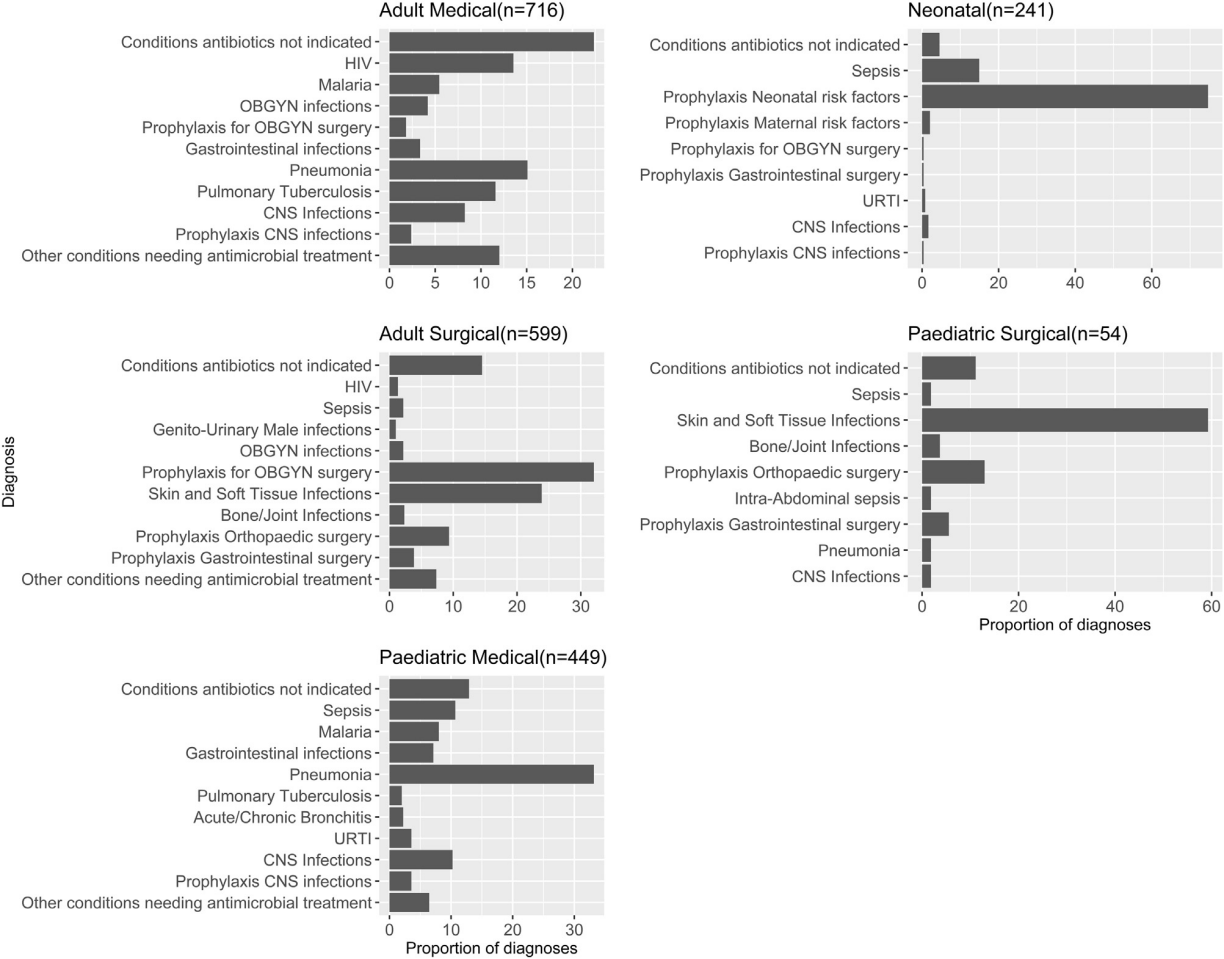
Bar Plot illustrating the top ten diagnoses and their proportions (%) for each department. n = total number of diagnoses in each department. CNS; Central Nervous System, URTI; Upper Respiratory tract infection, OBGYN; Obstetrics and Gynaecology.

There were 2059 diagnoses (adult medical = 716, adult surgical = 599, paediatric medical = 449, paediatric surgical = 54 and neonatal 241) recorded from the 1675 hospitalised patients. For adults in the medical units, the most frequent diagnostic category was “conditions for which antibiotics are not indicated” accounting for 160/716 (21%) of the diagnoses in that department. In paediatrics, the most common diagnosis was pneumonia or lower respiratory tract infections (148/449 [33%]). To give a clear picture of the disease patterns in these hospital departments, Figure 2 presents the top ten diagnoses for each department documented for the 1675 patients and therefore includes some parasitic (malaria) and viral infections like HIV. All other diagnoses in a department are collapsed into the category “other conditions needing antimicrobial treatment”. Where relevant the viral and parasitic infections were excluded from the analysis on antibiotic use.

### Guideline availability and treatment appropriateness

We identified four major local guidelines. A guide on management of common illnesses in level 4–6 hospitals (Ministry of Medical Services and Ministry of Public Health and Sanitation, 2009), national guidelines for the treatment of sexually transmitted illnesses (Ministry of Health, 2018), the basic paediatric protocol (Ministry of Health, 2016), and a local hospital guideline for use in the national referral hospital (Kenyatta National Hospital and University of Nairobi, 2018). Others were from the British Thoracic Society(1 condition), Infectious Diseases Society of America (10 conditions), the Surviving Sepsis Campaign(2 conditions) and the National Institute for Health and Care Excellence (2 conditions). Full list is provided in Supplement 5.

**Figure 3:**
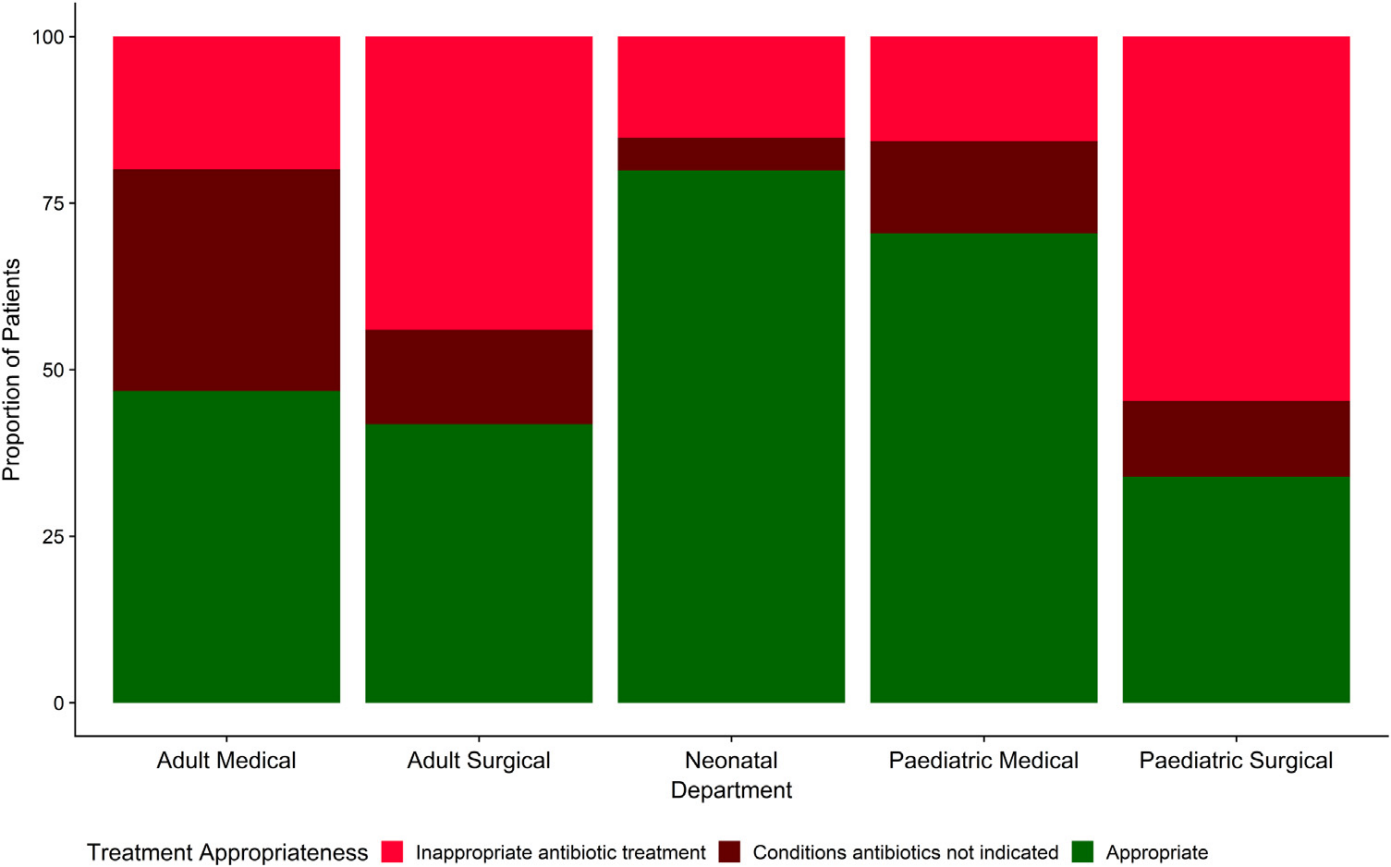
Bar chart showing the proportion (%) of patients receiving appropriate treatment by department based on 1502 patients with a single diagnosis.

There were 1502(90%) of the 1675 hospitalised patients,(Adult medical = 421; adult surgical = 543; paediatric medical = 261; paediatric surgical = 53 and neonatal = 224 patients), where we could ascertain a final diagnosis. This allowed for assessment of the per-patient appropriateness of treatment by condition. Overall, 805(53.6%) of the 1502 patients had appropriate treatment. The highest percentage of appropriate treatment was in the neonatal department at 80% [179/224]). Of the 224 patients in the adult medical unit whose treatment was inappropriate, 140 (63%) prescriptions were for conditions not requiring antibiotic treatment. This is shown in Figure 3

### Treatment appropriateness by disease conditions

**Figure 4:**
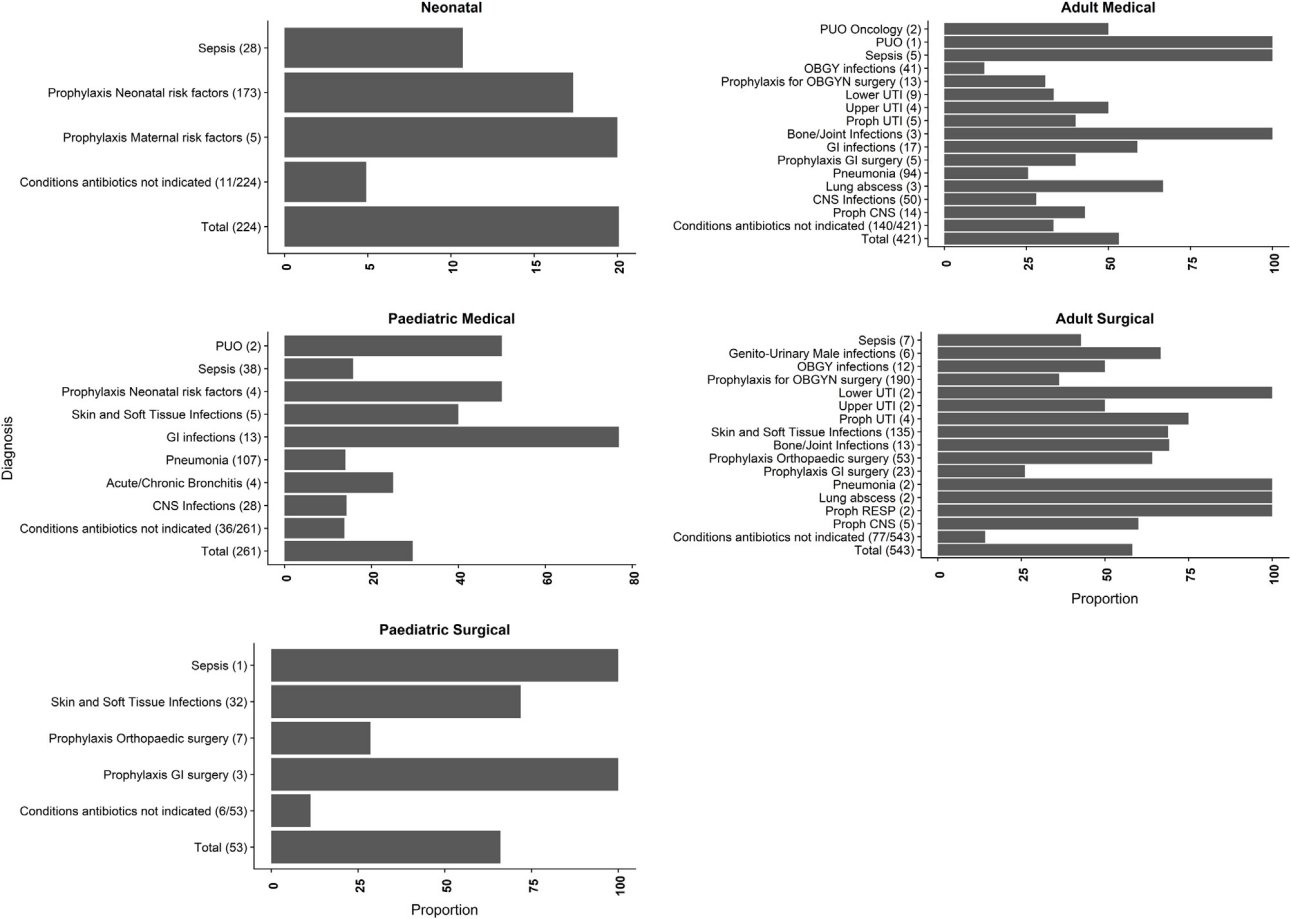
Proportion of treatment inappropriateness by disease conditions across the departments. The number on brackets () indicates the total number of patients with the disease. Patients with antibiotic treatment for conditions where antibiotics are not required are presented as a proportion of the total number of patients in the department. PUO- Pyrexia of unknown origin, UTI- Urinary Tract Infection, CNS-Central Nervous System, GI-Gastro-Intestinal, OBGYN Obstetrics and Gynaecology.

We further explored the disease conditions that were treated appropriately by the departments from these 1502 patients where a single final diagnosis was assigned. In the adult medical department, 24 (26%) of the 94 patients with pneumonia had inappropriate treatment. In the adult surgical unit, skin and soft tissue infections formed a large proportion of patients, 93/135 (68%) received inappropriate treatment. This is shown in Figure 4. Patients with a final diagnosis of conditions where antibiotic treatment was not indicated are presented as a proportion of the total number of patients.

### Physical availability of guidelines

We assessed which treatment guidelines were physically available during the survey (Supplement 6). The only physically available guideline was the basic paediatric protocols. These guidelines cover ten conditions, five in each of the paediatrics and neonates. The five conditions for which guidelines were available in the neonatal and paediatric units accounted for 94% (210/224) and 56%(147/261) of the patients with a final diagnosis respectively.

### Factors associated with treatment appropriateness

We assessed the main factors that may be associated with treatment appropriateness in both univariate models and a multiple regression model. These were duration of hospital stay since admission, gender, physical availability of guidelines and number of diagnoses.

We ran these models considering two possible outcomes, I. Overall treatment appropriateness for all patients on antibiotics and II; appropriateness only for patients with conditions requiring antibiotics.

Both outcomes resulted in the same general pattern of results. With overall treatment appropriateness for all patients on antibiotics as the outcome, there was evidence of a negative association of appropriateness of treatment with increasing the duration of hospital stay. Quadratic terms for the duration of stay were not statistically significant; hence a linear relationship was assumed. Physical availability of treatment guidelines increased the odds of receiving appropriate treatment. There was an increase in the odds of appropriate treatment with an increase in the number of diagnoses for the population of patients receiving antibiotics (Table 2).

**Table 2:** Univariate and multivariable models for factors influencing appropriate treatment for patients with conditions requiring antibiotic treatment and for all patients on antibiotics.

		Univariate Regression								Multiple regression							
Variable	level	Appropriate treatment for patients with conditions requiring antibiotics				Appropriate treatment for all patients receiving antibiotics				Appropriate treatment for patients with conditions requiring antibiotics				Appropriate treatment for all patients receiving antibiotics			
		OR	95%CI		p value	OR	95%CI		p value	OR	95%CI		p value	OR	95%CI		p value
Duration of hospital stay		0.63	0.55	0.71	<0.001	0.71	0.63	0.79	<0.001	0.65	0.57	0.74	<0.001	0.71	0.63	0.81	<0.001
Guidelines available in hospital	Yes	3.73	2.79	4.99	<0.001	6.34	4.78	8.41	<0.001	3.65	2.7	4.95	<0.001	6.44	4.81	8.64	<0.001
Number of diagnoses	1	Ref								Ref							
	2	1.12	0.8	1.57	0.671	1.33	0.99	1.8	0.013	1.08	0.76	1.55	0.334	1.28	0.92	1.79	0.005
	>2	1.21	0.68	2.15		1.98	1.12	3.51		1.58	0.84	2.99		2.58	1.37	4.87	
Gender	Male	0.98	0.77	1.24	0.859	1.09	0.88	1.34	0.429	0.84	0.65	1.09	0.198	0.84	0.67	1.07	0.154

## Discussion

From this report about half the hospitalised patients were on antimicrobial treatments, mainly antibiotics. The proportions of patients on antibiotics varied across hospital departments with almost three-quarters of the admitted paediatric medical patients on treatment. This prevalence of antibiotic use is consistent with other studies in Africa, with some studies reporting proportions higher than 70% among hospitalised patients (Mulwa et al., 2015, Chukwuani et al., 2002). Cephalosporins and penicillins accounted for a substantial portion of these prescriptions. A significant increase in the use of penicillins and cephalosporins, particularly ceftriaxone over the last decade was identified globally (Van Boeckel et al., 2014). This has been attributed to factors including economic growth, increased expenditure on health and increased access to medicines (Van Boeckel et al., 2014). The use of cephalosporins has also been increased by its convenient frequency of administration which may be advantageous on understaffed units and may have lower drug costs (purchase, preparation and administration) compared to other antibiotics that require multiple daily doses (Davis and Bryson, 1994).

From this survey, less than 1% of the antibiotic prescriptions were supported by laboratory data. Lack of laboratory support influences prescription patterns and choice (Chem et al., 2018). This results in most of the treatment being broad-spectrum, also encouraging polypharmacy, and may fuel drug resistance (Chokshi et al., 2019). Some challenges identified in laboratories have been, the high cost of investigations, long turnaround time and inaccurate results (Alemnji et al., 2014). Improving laboratory capacity to conduct tests like cultures and antibiotic susceptibility testing would translate to increase costs of care for the patient. The use of regional referral laboratories supported by governments to carry out such tests may mean lower costs for patients due to economies of scale (Ministry of Health, 2014). These extra laboratory costs need to be viewed in light of the unnecessary costs incurred due to unwarranted treatment and hospital stay in cases where treatment is not supported by laboratory data.

Our data suggest an association between the physical availability of treatment guidelines and appropriate treatment. It is striking that across 14 hospitals, guidelines were only physically available on paediatric and neonatal units. Better access to approved guidelines for common illnesses is imperative. We note that the availability and use of the paediatric guidelines have been supported by other interventions which include nationwide dissemination of the guidelines by the Ministry of Health and the training of clinicians in hospitals and universities on the use of these guidelines through the emergency treatment and triage programme (English et al., 2017b; Irimu et al., 2008). Adherence to locally relevant treatment guidelines may improve patient outcomes by improving mortality, reducing the length of hospital stay and readmissions (Wathne et al., 2019). Although we assessed for physical availability of guidelines, we acknowledge that some of the international guidelines are also available electronically. Availing local guidelines electronically may increase usage due to the extensive internet coverage in Kenya.

In addition to the hospital level factors like laboratory and guideline availability, clinician level factors may also affect antibiotic prescription. Level of training and clinical experience is crucial in decision making and guideline adherence (Ogero et al., 2020). Most of the hospitals in our study were internship training centres. Hence, prescriptions were done by different cadres of clinicians with varying levels of training. Though data on prescribers were not collected in this study, work in similar settings indicates that more than 85% of prescriptions in the paediatric units are done by junior clinicians mainly in the pre-registration period of training (Ogero et al., 2020). Therefore, in addition to providing guidelines, improving supervision of the junior clinicians is crucial (Papoutsi et al., 2017). Other interventions that could enhance proper antibiotic use include functional antibiotic stewardship committees that can audit antibiotic use and provide feedback to the clinicians (Wei et al., 2017).

Our data collection included Mondays in deviation to the Global-PPS recommendation, especially on surgical prophylaxis data (GLOBAL-PPS, 2015). This was because patients in our hospitals were admitted for elective surgeries throughout the week. Additional analysis showed no differences in the prescriptions patterns and appropriateness by day of the week. Using the PPS approach has some limitations. It does not capture seasonal variations in disease patterns, treatment outcomes and does not establish which antibiotics were available in the hospitals, which may influence the prescriptions. Additionally, other investigations including, radiology and haematology, which may have informed the antibiotic prescriptions are not considered in this PPS approach.

We acknowledge the clinical specialties used to assess appropriateness did not include clinical microbiologists and pharmacists. While including them would have been ideal, we consulted an infectious disease specialist to assist where the decisions on appropriateness were not clear. Additionally, the guidelines we included provided different treatment approaches for similar illnesses hence accommodating the differences in antibiotic selection between the prescribers.

Our assessment of appropriateness did not consider the doses and duration of treatment. However, we note that in more than 96% of the prescriptions, the dose and duration were documented. Finally, this study was performed in public hospitals, although these are the majority, it does not inform about appropriateness of prescription and usage in private hospitals in Kenya.

## Conclusion

We report that 46.7% of all inpatients were on antimicrobial treatment, mainly antibiotics. In <0.1% of patients, the antibiotic prescription was supported by laboratory tests. Many patients did not have a recorded diagnosis that warranted an antibiotic prescription, and there is a strong indication that physical availability of guidelines may influence treatment appropriateness positively. This situation may be improved by availing treatment guidelines across all the departments and providing better diagnostic support and training for clinicians.

## Authors' contributions

The roles of the contributors were as follows: M.M, J.M, O.T, C.S and M.E conceived the study. M.M E.O and N.K reviewed the prescription data. M.M and P.M conducted data analysis. M.M, J.M, O.T, C.S and M.E drafted and critically revised the manuscript for intellectual content. All authors read and approved the final manuscript.

## Funding

This work was supported by funds from the economic and social research council ESRCS #ES/P004938/1, and a Senior Research Fellowship awarded to ME by The 10.13039/100010269Wellcome Trust (#207522). MM is supported by a grant from by the Initiative to Develop African Research Leaders (IDeAL) through the DELTAS Africa Initiative [DEL-15-003], an independent funding scheme of the African Academy of Sciences (AAS)'s Alliance for Accelerating Excellence in Science in Africa (AESA) and supported by the New Partnership for Africa's Development Planning and Coordinating Agency (NEPAD Agency) with funding from the Wellcome Trust [107769/Z/10/Z] and the UK government. The funders had no role in drafting nor the decision for submitting this manuscript.

## Conflict of interest

None.

## Ethical approval

This study received approval from the Oxford Tropical research ethics committee (OXTREC) from the University of Oxford (Ref: 525–17) and the Kenyan Medical Research Institute (Ref: KEMRI/SERU/CGMR-C//086/3450).

## Availability of data and materials

Data used for this manuscript are available in Harvard Dataverse at https://dataverse.harvard.edu/dataset.xhtml?persistentId=doi:10.7910/DVN/L7S8TK. Applications for access can be made through the Data Governance Committee with details available on www.kemri-wellcome.org, or on email to cgmrc@kemri-wellcome.org.

## References

[bib0005] Ahoyo T.A., Bankolé H.S., Adéoti F.M., Gbohoun A.A., Assavèdo S., Guénou M.A. (2014). Prevalence of nosocomial infections and anti-infective therapy in Benin: results of the first nationwide survey in 2012. Antimicrob Resist Infect Control.

[bib0010] Alemnji G.A., Zeh C., Yao K., Fonjungo P.N. (2014). Strengthening national health laboratories in sub-Saharan Africa: a decade of remarkable progress. Trop Med Int Health.

[bib0015] Apat D.O., Gachohi J.M., Karama M., Kiplimo J.R., Sachs S.E. (2017). Temporal variation in confirmed diagnosis of fever-related malarial cases among children under-5 years by community health workers and in health facilities between years 2013 and 2015 in Siaya County, Kenya. Malar J.

[bib0020] Ayukekbong J.A., Ntemgwa M., Atabe A.N. (2017). The threat of antimicrobial resistance in developing countries: causes and control strategies. Antimicrob Resist Infect Control.

[bib0025] Chem E.D., Anong D.N., Akoachere J.K.T. (2018). Prescribing patterns and associated factors of antibiotic prescription in primary health care facilities of Kumbo East and Kumbo West Health Districts, North West Cameroon. PLoS One.

[bib0030] Chokshi A., Sifri Z., Cennimo D., Horng H. (2019). Global Contributors to Antibiotic ResistanceGlobal contributors to antibiotic resistance. J Glob Infect Dis.

[bib0035] Chukwuani C.M., Onifade M., Sumonu K. (2002). Survey of drug use practices and antibiotic prescribing pattern at a general hospital in Nigeria. Pharm World Sci.

[bib0040] Davis R., Bryson H.M. (1994). Ceftriaxone. PharmacoEcon.

[bib0045] Doron S., Davidson L.E. (2011). Antimicrobial stewardship. Mayo Clin Proc.

[bib0050] Elbireer A.M., Jackson J.B., Sendagire H., Opio A., Bagenda D., Amukele T.K. (2013). The good, the bad, and the unknown: quality of clinical laboratories in Kampala, Uganda. PLoS One.

[bib0055] English M., Ayieko P., Nyamai R., Were F., Githanga D., Irimu G. (2017). What do we think we are doing? How might a clinical information network be promoting implementation of recommended paediatric care practices in Kenyan hospitals?. Health Res Policy Syst.

[bib0060] English M., Irimu G., Nyamai R., Were F., Garner P., Opiyo N. (2017). Developing guidelines in low-income and middleincome countries: lessons from Kenya. Arch Dis Childhood.

[bib0065] Gharbi M., Doerholt K., Vergnano S., Bielicki J.A., Paulus S., Menson E., ARPEC project Group (2016). Using a simple point-prevalence survey to define appropriate antibiotic prescribing in hospitalised children across the UK. BMJ Open.

[bib0070] GLOBAL-PPS (2015). Global Point Prevalence Survey of Antimicrobial Consumption and Resistance. http://www.global-pps.com/.

[bib0075] Harris P.A., Taylor R., Thielke R., Payne J., Gonzalez N., Conde J.G. (2009). Research electronic data capture (REDCap)–a metadata-driven methodology and workflow process for providing translational research informatics support. J Biomed Inform.

[bib0080] Irimu G., Wamae A., Wasunna A., Were F., Ntoburi S., Opiyo N. (2008). Developing and introducing evidence based clinical practice guidelines for serious illness in Kenya. Arch Dis Child.

[bib0085] Kenyatta National Hospital and University of Nairobi (2018). The KNH Guide to Empiric Antimicrobial Therapy.

[bib0090] Maina M., Auguet O.T., McKnight J., Zosi M., Kimemia G., Mwaniki P. (2019). Evaluating the foundations that help avert antimicrobial resistance: performance of essential water sanitation and hygiene functions in hospitals and requirements for action in Kenya. PLoS One.

[bib0095] Ministry of Environment and Forestry, State Department of Environment (2018). Review of Rainfall During the March to May 2018 “Long Rains” Season and the Outlook for the June-July-August (jja) 2018.

[bib0100] Ministry of Health (2016). Basic Paediatric Protocols for Ages up to 5 Years.

[bib0105] Ministry of Health (2018). Kenya National Guidelines for Prevention, Management and Control of Sexually Transmitted Infections.

[bib0110] Ministry of Health, Division of Emergency, Disaster Risk Management (2014). Kenya Health Sector Referral Strategy (2014–2018).

[bib0115] Ministry of Medical Services and Ministry of Public Health and Sanitation (2009). Clinical Management and Referral Guidelines – Volume III: Clinical Guidelines for Management and Referral of Common Conditions at Levels 4–6: Hospitals.

[bib0120] Mulwa N.C., Osajo G.O., Ndwigah S.N., Kaburi A.N., Muriuki G. (2015). Patterns of prescribing practices in makueni county referral hospital, Kenya. Afr J Pharmacol Ther.

[bib0125] Nobili A., Licata G., Salerno F., Pacina L., Tettamanti M., Franchi C. (2011). SIMI Investigators. Polypharmacy, length of hospital stay, and in-hospital mortality among elderly patients in internal medicine wards. The REPOSI study. Eur J Clin Pharmacol.

[bib0130] O’neill J. (2014). Antimicrobial resistance: tackling a crisis for the health and wealth of nations. Rev Antimicrob Resist.

[bib0135] Ogero M., Akech S., Malla L., Agweyu A., Irimu G., English M., Clinical Information Network Author Group (2020). Examining which clinicians provide admission hospital care in a high mortality setting and their adherence to guidelines: an observational study in 13 hospitals. Arch Dis Childhood.

[bib0140] Okoth C., Opanga S., Okalebo F., Oluka M., Kurdi A.B., Godman B. (2018). Point prevalence survey of antibiotic use and resistance at a referral hospital in Kenya: findings and implications. Hosp Pract (1995).

[bib0145] Papoutsi C., Mattick K., Pearson M., Brennan N., Briscoe S., Wong G. (2017). Social and professional influences on antimicrobial prescribing for doctors-in-training: a realist review. J Antimicrob Chemother.

[bib0150] Paterson D.L. (2006). The Role of antimicrobial management programs in optimizing antibiotic prescribing within hospitals. Clin Infect Dis.

[bib0155] Petti C.A., Polage C.R., Quinn T.C., Ronald A.R., Sande M.A. (2006). Laboratory medicine in Africa: a barrier to effective health care. Clin Infect Dis.

[bib0160] Pinheiro J.C., Bates D.M., Lindstrom M.J. (1995). Model Building for Nonlinear Mixed Effects Models.

[bib0165] R Core Team (2013). R: A Language and Environment for Statistical Computing.

[bib0170] Republic of Kenya (2017). National Policy for the Prevention and Containment of Antimicrobial Resistance.

[bib0175] Tornheim J.A., Manya A.S., Oyando N., Kabaka S., O’Reilly C.E., Breiman R.F. (2010). The epidemiology of hospitalization with diarrhea in rural Kenya: the utility of existing health facility data in developing countries. Int J Infect Dis.

[bib0180] Van Boeckel T.P., Gandra S., Ashok A., Caudron Q., Grenfell B.T., Levin S.A. (2014). Global antibiotic consumption 2000 to 2010: an analysis of national pharmaceutical sales data. Lancet Infect Dis.

[bib0185] Versporten A., Zarb P., Caniaux I., Gros M.F., Drapier N., Miller M. (2018). Global-PPS network. Antimicrobial consumption and resistance in adult hospital inpatients in 53 countries: results of an internet-based global point prevalence survey. Lancet Global Health.

[bib0190] Wathne J.S., Harthug S., Kleppe L.K.S., Blix H.S., Nilsen R.M., Charani E. (2019). The association between adherence to national antibiotic guidelines and mortality, readmission and length of stay in hospital inpatients: results from a Norwegian multicentre, observational cohort study. Antimicrob Resist Infect Control.

[bib0195] Wei X., Zhang Z., Walley J.D., Hicks J.P., Zeng J., Deng S. (2017). Effect of a training and educational intervention for physicians and caregivers on antibiotic prescribing for upper respiratory tract infections in children at primary care facilities in rural China: a cluster-randomised controlled trial. Lancet Global Health.

[bib0200] World Health Organization (2013). Pocket Book of Hospital Care for Children: Guidelines for the Management of Common Childhood Illnesses.

[bib0205] World Health Organization (2015). Guidelines for ATC Classification and DDD Assigment.

