# Supplementary material for: Antibiotic use in Kenyan public hospitals: Prevalence, appropriateness and link to guideline availability
Source: Int J Infect Dis. 2020 Oct;99:10–8. doi: 10.1016/j.ijid.2020.07.084 (PMC7562818; doi:10.1016/j.ijid.2020.07.084)

**Supplement 1: Hospital capacity and population demographics**

| Facility | Hospital Bed capacity | Number of specialist doctors  (consultants) | Number of wards in the facility | County HIV Prevalence ^a^ | Catchment population ^b^ | Wards Excluded by Specialty |
| --- | --- | --- | --- | --- | --- | --- |
| High Malaria Prevalence Zone | | | | | | |
| H1 | 204 | 5 | 7 | 7.7% | 142,408 | Renal Unit |
| H3 | 550 | 12 | 14 | 4.5 | 188,212 | Psychiatry, Renal Unit, ENT |
| H7 | 180 | 7 | 6 | 16.3% | 220,997 | None |
| H8 | 250 | 14 | 8 | 4.3% | 147,992 | Renal Unit |
| H14 | 165 | 5 | 5 | 5.4 | 95,292 | None |
| Low Malaria prevalence Zone | | | | | | |
| H2 | 594 | 26 | 16 | 2.8 | 127,100 | ENT, ICU, Psychiatry, Renal |
| H4 | 216 | 8 | 7 | 3.7 | 99,065 | None |
| H5 | 231 | 7 | 9 | 3.1 | 122,740 | Psychiatry |
| H6 | 383 | 17 | 10 | 4% | 145,903 | Neurosurgery |
| H9 | 550 | 19 | 18 | 3.8 | 170,606 | ENT, Renal, Psychiatry, ICU |
| H10 | 131 | 24 | 6 | 6.1 | 988,808 | None |
| H11 | 320 | 21 | 9 | 6.1 | 197,489 | None |
| H13 | 378 | 20 | 15 | 3.7 | 140,338 | ENT, Renal, Psychiatry, ICU, Eye |
| H16 | 350 | 14 | 6 | 6.1% | 268,276 | None |

a Kenyan Ministry of Health. "Kenya HIV estimates report 2018." (2018).

b 2019 Kenya Population and Housing Census: Distribution of Population by Administrative units [http://www.knbs.or.ke](http://www.knbs.or.ke/)

**Supplement 2: Ward Level Data Collection Tool**

| **Please fill in one form for each ward and department type identified within the ward** | | | | | |
| --- | --- | --- | --- | --- | --- |
| **Unique ID** | ____________________________________ | | | | |
| **Date of survey** (dd/mm/year) | ___________/________/________________ | | | | |
| **Auditor code** (Person completing form) | ____________________________________ | | |  | |
| **Hospital name** | ____________________________________ | | |  | |
| **Ward name** | **□** Adult Medical (1-6) | **□** Orthopaedics (1-6) | **□** Gynaecology (1-6) | | **□** Other:_____________________ |
|  | **□** Adult Surgical (1-6) | **□** Paediatrics (1-6) | **□** Maternity (1-6) | |  |
|  | **□** Surgical (1-6) | **□** Neonatal (1-6) | **□** Post-Natal (1-6) | |  |
|  |  |  | |  |  |
| **Does the ward have patients from mixed departments?** | **□** Yes **□** No |  | |  | |
| **Choose department type:** | **□** Adult Medical | **□** Paediatric Medical | | **□** Neonatal | |
|  | **□** Adult Surgical | **□** Paediatric Surgical | |  | |
|  |  |  | |  | |
| **Choose patient population for this department type** | □ Male | □ Female | | □ Paediatric / Neonatal | |
|  |  |  | |  | |
| **Total number of admitted patients present** on this ward, for the selected department type, and who were admitted before 8 am: ___________________ | | | | | |
|  | | | | | |
| **Total number of beds** on this ward for the selected department type, which were present at 8 am on the day of the survey: ___________________ | | | | | |
|  | | | | | |
| ***NOTES***   - Fill in one form for each ward and department type identified within the ward. - Date of Survey refers to the date for the current data abstraction – *click today on the calendar*. - 'Unique ID' is automatically generated by the REDCap when you click this field. This is a unique code used to identify each ward form record. - If the name of the ward is other than those listed, choose 'other' and enter ward name and number (i.e. either 1, 2, 3 …6) to identify this ward uniquely - Include only inpatients "admitted before 08:00 o'clock" on the day of the survey - For numerical fields, type -1 if the value is unknown - Orthopaedic and neurosurgery patients correspond to the surgical department type - DO NOT conduct a survey of the following wards: Amenity, Psychiatry, ENT, Renal, ICU, NICU (including in Kenyatta hospital), Neurosurgery and Eye - In the case of paediatrics and neonatal, beds may correspond to cots or incubators. Cots and incubators must be included in the total 'bed count' where applicable. | | | | | |

**Supplement 3: Patient-level data collection tool and diagnosis list**

| Please fill in one form per patient on antibiotic or other eligible antimicrobial treatment/prophylaxis—explanatory Notes I explains how to fill this form. |
| --- |
| \| Unique \| Patient IP \| Hospital Name \| Ward Name \| Activity \| Today's Date \| Admission Date \| Date of Surgery I \| Date of Surgery II \| Re-admission \| \| --- \| --- \| --- \| --- \| --- \| --- \| --- \| --- \| --- \| --- \| \| ID ^1^ \| Number ^2^ \| Name/Code \| Name/Code \| (Department Type) ^3^ \| --/--/----^4^ \| --/--/----^5^ \| --/--/---- ^6^ \| --/--/---- ^7^ \| Y □ / N □ / Empty □ ^8^ \| |
|  |
| \| Is Patient’s age documented? ^9^ \| Patient Age ^10^ : ≥ 2 years □ / 1-23 months □/ <1 month □ \| \| \| Weight ^11^ \| Gender ^12^ \| Diagnosis (Explanatory Note II) ^13^ \| \| \| --- \| --- \| --- \| --- \| --- \| --- \| --- \| --- \| \| Y □ / N □ \| Years  (if ≥ 2 years):  ____________ \| Months  (1-23 months):  _____________ \| Days  (if <1 month):  _____________ \| (in Kg; 1 decimal) \| □ M  □ F \| □ Diagnosis 1 \| □ Diagnosis 4 \| \| □ Diagnosis 2 \| □ Diagnosis 5 \| \| □ Diagnosis 3 \|  \| |
|  |
| \| Treatment based on biomarker data? ^14^ \| Y □ / N □ \| If yes, which biomarker? ^15^ \| CRP □ / PCT □ / Other □ \| Number of antimicrobials given ^18^ \| \| --- \| --- \| --- \| --- \| --- \| \| Type of biological fluid sample? ^16^ \| Blood □ / Urine □ / Other □ \| Most recent value of biomarker (mg/l)? ^17^ \| ____________________ \| (from 0 to 5) \| |
|  |
| \| Antibiotic or other antimicrobial name: ^19^  _____________________________ \| \| \| \| \| --- \| --- \| --- \| --- \| \| Start Date: ^20^ \| __/__/____ \| Type of indication (Explanatory note IV) ^25^ \| _____________________________ \| \| Single Unit Dose: ^21^ \| __________ \| Reason in notes ^26^ \| Y □ / N □ \| \| Unit: ^22^ \| g □ / mg □ / mega unit □ / tablet □/ IU □/ empty □ \| Guideline compliance ^27^ \| Y □ / N □ / NA □ / NI □ \| \| Doses per day: ^23^ \| OD □ / BD □ / TID □ / OID □/ empty □/ Other □:__________ \| Stop/Review date documented: ^28^ \| Y □ / N □ \| \| Route: ^24^ \| P □ / O □ / R □ / I □/ empty □/ Other □:__________ \| Duration: ^29^ \| _____________________________ \| |
|  |
| \| Treatment (E: Empirical / T: Targeted) ^30^ \| E □ / T □ \| *The next section is to be filled in only if the treatment of choice is based on microbiology data (i.e. treatment = targeted):* \| \| \| --- \| --- \| --- \| --- \| \| MRSA ^31^ \| \| \| Y □ / N □ \| \| MRConS ^32^ \| \| \| Y □ / N □ \| \| VRE ^33^ \| \| \| Y □ / N □ \| \| ESBL-producing enterobacteriaceae ^34^ \| \| \| Y □ / N □ \| \| Third generation cephalosporin resistant enterobacteriaceae / non-ESBL or ESBL status unknown ^35^ \| \| \| Y □ / N □ \| \| Carbapenem-resistant enterobacteriaceae ^36^ \| \| \| Y □ / N □ \| \| ESBL-producing non-fermenter Gram negative bacilli ^37^ \| \| \| Y □ / N □ \| \| Carbapenem-resistant non-fermenter Gram negative bacilli ^38^ \| \| \| Y □ / N □ \| \| Targeted treatment against other MDR organism ^39^ \| \| \| Y □ / N □ \| |

***Explanatory Notes I – Patient Form***

*Adapted from the Global Point Prevalence Survey (2017 GLOBAL-PPS): http://www.global-pps.com/documents*

1. Unique ID: This is a 'survey number', a unique non-identifiable number given by the electronic system for each patient entered in the database. Note down this number with the corresponding 'Patient IP Number' every time you enter patient information in the database. This will allow you to retrieve the record for this patient again; in the event, you need to amend information or record additional information. You can only search patients in the database, using the survey number. The unique ID is a seven-digit auto-generated and incremental number. For example; Kiambu, the first record would read *5100001*.
2. Patient IP Number: A unique inpatient identifier that allows linkage to patient records at the local level for a more detailed audit. This unique identifier will eventually be removed from the database after the data has been cleaned, and before the analysis starts. Check all the medical record notes, including file covers, for the IP number.
3. Activity: This is the department type. E.g. M=medicine, S=surgery (including orthopaedics, and any other surgical patients in obstetrics or other), etc.
4. Today's Date: date when records are abstracted. Click on 'today' icon to generate the date for today.
5. Admission Date: Please indicate the date of admission as indicated on the ward clinical admission notes- it is the date when the patient is first seen by a clinician. If not available check in other sections of the file (e.g. cardex) or ask the ward staff.
6. Date of Surgery I: This question is only applicable for patients in a surgical department. If a surgical department was indicated under activity, this section will appear in the electronic system. Please indicate the date when the patient first underwent surgery during the current admission, as indicated on any of the clinical surgical notes. If not available check in other sections of the file (e.g. cardex, theatre notes). If date of surgery is definitely missing, enter 01/01/1913
7. Date of Surgery II: As above, if a surgical department was indicated under activity, this section will appear. Please indicate the date when the patient underwent subsequent surgery during the current admission, as indicated on any of the clinical surgical notes. If not available check in other sections of the file (e.g. cardex, theatre notes). If the patient underwent subsequent surgery but the date is definitely missing, enter 01/01/1913. If the patient did not undergo subsequent surgery, enter 01/01/1914. This means 'not applicable'
8. Re-admission: Refers to re-admission to this hospital within the 6 months preceding today's date. Please indicate yes if the patient has been admitted in this hospital within the previous 6 months. You can check for this information in the discharge summary for the previous admission^.^ If not documented, enter Empty.
9. Is patient's age documented? Enter yes or no. If yes, a drop box prompting to specify the age group will appear in the system.
10. Patient's Age: Choose whether patient is <1 month; 1-23 months old or ≥ 2 years old. If the patient is 2 years old or older, the system will ask you to enter age in years; if between 1 and 23 months you will be asked to enter the number of months (e.g. 1 to 23), if less than 1 month you will be ask to enter the number of days. To report age in years, round to the lowest number (e.g. 2 years and one month = 2 years; 2 years and 11 months = 2 years). If the information is not available, enter -1.
11. Weight: This refers to admission weight. Round off weight to the nearest one decimal place e.g. 6.750 kg = 6.8 kg. Check from the admission notes. If missing, check on the treatment sheet or nursing cardex. If two readings for the admission day are different, enter the one on the treatment sheet. If the weight is not documented, please indicate -1.
12. Gender: Please check from the admission notes. If not documented check from the records registration form. Alternatively, you can ask a member of staff to check on the gender of the patient, as he/she will be physically in the ward.
13. Diagnosis/ses: These are what clinicians aim to treat. See diagnoses groups list in Explanatory Notes II. You may enter up to five diagnoses by order of priority (i.e. diagnosis 1 would correspond to the main or most severe condition). You must then select the diagnosis group (as shown in Explanatory Notes II), that best describes the diagnosis recorded in the patient's clinical notes.
14. Treatment based on biomarker data: Tick yes if CRP (C-reactive protein), PCT (Procalcitonin) or culture results are available in clinical notes for this patient. Click no if none of these laboratory tests were conducted for this patient.
15. If yes, which biomarker: If treatment based on biomarker, specify which one: CRP (C-reactive protein), PCT (Procalcitonin) or Other (=lab-based culture and sensitivity result from a relevant biological sample). You may choose more than one option.
16. Type of biological fluid sample: Indicate which type of sample was used to measure the biomarker (either blood, urine or other [e.g. cerebrospinal fluid, pus, etc.]). You may choose more than one option.
17. Most recent value of biomarker (mg/l): If CRP (C-reactive protein) or PCT (Procalcitonin) results are available, write the value of the latest test result in mg/l.
18. Number of antimicrobials given: Enter the total number of antimicrobials given to the patient, counting only those antimicrobial treatments that meet the eligibility criteria described in the outline protocol. If no antimicrobials were given to the patient, enter zero.
19. Antibiotic or other antimicrobial name: Select the generic antibiotic or antimicrobial name from the dropdown list. Please note the dropdown list does not display brand names. You must find out the generic name in case a brand name is noted in the clinical files instead.
20. Start date: Indicate the date when the treatment started. If this is not available, enter 01/01/1913
21. Single Unit Dose: Numeric value for dose per administration (in grams, milligrams, megaunits, tablets or IU). Please see Explanatory Notes III for reporting specific drug combinations. If dose is missing enter -1.
22. Unit: The unit for the dose (g, mg, mg, tablet or IU) as documented in the treatment sheet. Indicate empty if not documented.
23. Doses per day: This is the number of times given per day as per the dropdown list provided in the data capture system, and as documented by clinicians in the treatment charts or clinical notes (e.g. OD/once a day/24hrly or BD/twice a day/12hrly or TID/TDS/three times a day/8hrly or QID/4quarterly/6hrly or STAT [i.e. once only]).If the doses are not as described above choose 'other' from the dropdown list, and enter the dose as free text. If necessary provide fractions of doses: (e.g., every 16h = 1.5 doses per day, every 36h = 0.67 doses per day, every 48h = 0.5 doses per day). If doses are not documented at all choose empty.
24. Route: Indicate the route of administration as documented by the clinician. Routes of administration are: Parenteral (P [e.g. iv, im]), Oral (O), Rectal (R), Inhalation (I). Indicate empty if not documented. If route of administration is none of the above choose 'other' from the dropdown list and enter route as free text.
25. Type of Indication: Based on diagnosis and/or clinical signs and symptoms documented in clinical notes, choose the indicator that best describes the indication for the current antimicrobial treatment., See Indication codes in Explanatory Notes IV.
26. Reason in Notes**:** Indicate whether the diagnosis / indication for treatment is recorded in the patient's documentation (treatment chart, notes, etc.) at the start of antibiotic or other antimicrobial treatment (Yes or No)
27. Guideline Compliance: Refers to whether the antibiotic or antimicrobial choice (not route, dose, duration etc) follows local guidelines (Y: Yes; N: No; NA: Not assessable because no local guidelines for the specific indication; NI: no information because indication is unknown)
28. Stop/Review date documented: Choose 'Yes' if a stop review date is documented or if the duration of the treatment is indicated in the treatment sheet or the clinical notes. If the duration of treatment is indicated, this will be indicated as number of days or weeks or in the following form: X/7, X/52, etc. Choose 'No' if neither a stop/review date nor duration of treatment are documented.
29. Duration: Corresponds to the number of days for which the treatment has been prescribed, as shown by the number of days ticked on the treatment sheet (i.e. intended duration), even if not all prescribed doses have been given or if doses have been missed. If duration is not documented enter -1.
30. Treatment Type: Indicate whether the treatment of choice is supported by microbiology data (i.e. culture results and drug susceptibility test [DST] results available). If culture results and DST are available, indicate that the treatment is Targeted (T), if not indicate the treatment is Empirical (E). If Targeted, a list of organisms will appear, and you will need to indicate which of the organisms were identified, by ticking 'Yes' or 'No' for each organism listed:
31. Methicillin-resistant *Staphylococcus aureus* (MRSA)
32. Methicillin-resistant coagulase negative staphylococci (MRCoNS)
33. Vancomycin-resistant enterococci (VRE)
34. Bacteria, producing extended-spectrum beta-lactamases (ESBL)
35. Third generation cephalosporin resistant enterobacteriaceae / non-ESBL or ESBL status unknown
36. Carbapenem-resistant *Enterobacteriaceae* (CRE) – enteric bacteria resistant to imipenem, meropenem or other carbapenems
37. Nonfermenters: *Pseudomonas aeruginosa, Acinetobacter baumannii, Burkholderia spp., Stenotrophomonas maltophilia*
38. Carbapenem-resistant Nonfermenters (CR-NF) – nonfermenters resistant to imipenem, meropenem or other carbapenems
39. Multi-drug resistant (MDR) pathogens, others than the listed above.

***Explanatory Notes II – Diagnostic Codes***

*Adapted from the Global Point Prevalence Survey (2017 GLOBAL-PPS): http://www.global-pps.com/documents*

*Diagnostic Codes (What the Clinician Aims at Treating)*

| **Site** | **Codes** | **Examples** |
| --- | --- | --- |
|  |  |  |
| *CNS* | *Proph CNS* | Prophylaxis for CNS (neurosurgery, meningococcal) |
|  | *CNS* | Infections of the **C**entral **N**ervous **S**ystem |
| *EYE* | *Proph EYE* | Prophylaxis for Eye operations |
|  | *EYE* | Therapy for Eye infections e.g., Endophthalmitis |
| *ENT* | *Proph ENT* | Prophylaxis for **E**ar, **N**ose, **T**hroat (**Surgical or Medical prophylaxis=SP/MP**) |
|  | *ENT* | Therapy for **E**ar, **N**ose, **T**hroat infections including mouth, sinuses, larynx |
| *RESP* | *Proph RESP* | Pulmonary surgery, prophylaxis for **Resp**iratory pathogens e.g. for aspergillosis |
|  | *LUNG* | Lung abscess including aspergilloma |
|  | *URTI* | **U**pper **R**espiratory **T**ract viral **I**nfections including influenza but not ENT |
|  | *Bron* | Acute **Bron**chitis or exacerbations of chronic bronchitis |
|  | *Pneu* | **Pneu**monia or LRTI (lower respiratory tract infections) |
|  | *TB* | Pulmonary TB (Tuberculosis) |
| *CVS* | *Proph CVS* | **C**ardiac or **V**ascular Surgery, endocarditis prophylaxis |
|  | *CVS* | **C**ardio**V**ascular **S**ystem infections: endocarditis, endovascular prosthesis or device e.g.  pacemaker, vascular graft |
| *GI* | *Proph GI* | Surgery of the **G**astro-**I**ntestinal tract, liver or biliary tree, GI prophylaxis in neutropaenic  patients or hepatic failure |
|  | *GI* | GI infections (salmonellosis, *Campylobacter*, parasitic, *C.difficile*, etc.) |
|  | *IA* | **I**ntra-**A**bdominal sepsis including hepatobiliary, intra-abdominal abscess *etc*. |
| *SSTBJ* | *Proph BJ* | Prophylaxis for SST, for plastic or orthopaedic surgery (**B**one or **J**oint) |
|  | *SST* | **S**kin and **S**oft Tissue: Cellulitis, wound including surgical site infection, deep soft tissue |
|  |  | not involving bone e.g., infected pressure or diabetic ulcer, abscess |
|  | *BJ* | **B**one/**J**oint Infections: Septic arthritis (including prosthetic joint), osteomyelitis |
| *UTI* | *Proph UTI* | Prophylaxis for urological surgery **(SP)** or recurrent **U**rinary **T**ract **I**nfection **(MP)** |
|  | *Cys* | Lower UTI |
|  | *Pye* | Upper UTI including catheter related urinary tract infection, pyelonephritis |
| *GUOB* | *Proph OBGY* | Prophylaxis for **OB**stetric or **GY**naecological surgery |
|  | *OBGY* | **Ob**stetric/**Gy**naecological infections, **S**exual **T**ransmitted **D**iseases (**STD**) in women |
|  | *GUM* | **G**enito-**U**rinary **M**ales + Prostatitis, epididymo-orchitis, STD in men |
| *No defined site (NDS)* | *BAC* | Bacteraemia with no clear anatomic site and no shock |
|  | *SEPSIS* | Sepsis, sepsis syndrome or septic shock with no clear anatomic site |
|  | *Malaria* |  |
|  | *HIV* | Human immunodeficiency virus |
|  | *PUO* | **P**yrexia of **U**nknown **O**rigin - Fever syndrome with no identified source or site of infection |
|  | *PUO-HO* | Fever syndrome in the non-neutropaenic **H**aematology–**O**ncolgy patient with no  identified source of pathogen |
|  | *FN* | **F**ever in the **N**eutropenic patient |
|  | *LYMPH* | Infection of the **lymph**atics as the primary source of infection e.g. suppurative lymphadenitis |
|  | *conditions antibiotics not required* | Antibiotic prescribed with documentation for which there is no above diagnosis group |
|  | *MP-GEN* | Drug is used as **M**edical **P**rophylaxis in **gen**eral, without targeting a specific site, e.g.  antifungal prophylaxis during immunosuppression |
|  |  |  |
|  | *PROK* | Antimicrobial (e.g. erythromycin) prescribed for **Prok**inetic use |
| *Neonatal* | *MP-MAT* | Drug is used as Medical Prophylaxis for MATERNAL risk factors e.g. maternal prolonged rupture of membranes |
|  | *NEO-MP* | Drug is used as **M**edical **P**rophylaxis for **NEONATE** risk factors e.g. VLBW (Very Low  Birth Weight) and IUGR (Intrauterine Growth Restriction) |

***Explanatory Notes III – Patient Form***

*Adapted from the Global Point Prevalence Survey (2017 GLOBAL-PPS): http://www.global-pps.com/documents*

1. *Combinations of an antibiotic and an enzyme inhibitor*
2. Ampicillin and enzyme inhibitor: report only ampicillin dose (J01CR01)
3. Amoxicillin and enzyme inhibitor: report only amoxicillin dose (J01CR02)
4. Ticarcillin and enzyme inhibitor: report only ticarcillin dose (J01CR03)
5. Piperacillin and enzyme inhibitor: report only piperacillin dose (J01CR05)
6. Imipenem and enzyme inhibitor: report only imipenem dose (J01DH51)
7. Panipenem and betamipron: report only panipenem (J01DH55)

*Example:*

- Augmentin® 1.2g IV: 1g (amoxicillin) + 200mg (clavulanic acid), report only 1 g
- Piperacillin® 4.5g IV: 4g (piperacillin) + 500mg (tazobactam), report only 4 g

1. *Other combinations of multiple antimicrobial substances:*
2. J01EE01 Sulfamethoxazole and Trimethoprim: report the total amount of sulfamethoxazole and trimethoprim

*Example:*

- Co-trimoxazole 960mg: (sulfamethoxazole. 800mg + trimethoprim 160mg), report 960mg

Further information on agents included for the Global-PPS is available in the antimicrobial list. Only antimicrobial substance name need to be written down, NOT the ATC codes (excel file - available at website under documents: Global-PPS_antimicrobial_list.xlsx)

http://www.global-pps.com/

***Explanatory Notes IV – Patient Form***

*Adapted from the Global Point Prevalence Survey (2017 GLOBAL-PPS): http://www.global-pps.com/documents*

*(Type of Indications: Select one for each antibiotic or other antimicrobial)*

| *CAI: Community acquired infection* |  | *CAI* | Symptoms start <48 hours from admission to hospital (or present on admission). |
| --- | --- | --- | --- |
|  |  |  |  |
| *HAI: Healthcare associated infection* |  | *-* | Symptoms start ≥ 48 hours after admission to hospital. |
|  |  |  |  |
|  |  | *HAI1* | Post-operative surgical site infection (within: 30 days of surgery OR; 1 year after implant surgery) |
|  |  |  |  |
|  |  | *HAI2* | Intervention related infections including CR-BSI, VAP and C- UTI |
|  |  |  |  |
|  |  | *HAI3* | *C. difficile* associated diarrhoea (CDAD) (>48 h post- admission or <30 days after discharge from previous admission episode). |
|  |  |  |  |
|  |  | *HAI4* | Other hospital acquired infection (includes HAP, etc) |
|  |  |  |  |
|  |  | *HAI5* | Infection present on admission from another hospital (patient with infection from another hospital) |
|  |  |  |  |
|  |  | *HAI6* | Infection present on admission from long-term care facility (LTCF) or Nursing Home*. |
|  |  |  |  |
| *SP: Surgical prophylaxis* |  | *SP1* | Single dose |
|  |  | *SP2* | One day |
|  |  | *SP3* | >1 day |
|  |  |  |  |
| *MP: Medical prophylaxis* |  | *MP* | For example long term use to prevent UTI's or use of antifungals in patients undergoing chemotherapy or penicillin in asplenic patients, *etc*. |
|  |  |  |  |
| *OTH: Other* |  | *OTH* | For example erythromycin as a motility agent (motilin agonist). |
|  |  |  |  |
| *UNK: Unknown* |  | *UNK* | Completely unknown indication |

CR-BSI = Catheter Related-Blood Stream Infection; C-UTI = Catheter related-Urinary Tract Infection; HAP = Hospital Acquired Pneumonia; VAP = Ventilator Associated Pneumonia.

* Long-term care facilities represent a heterogeneous group of healthcare facilities, with care ranging from social to medical care. These are places of collective living where care and accommodation is provided as a package by a public-agency, non-profit or private company (e.g. nursing homes, residential homes).

Supplement 4

Antibiotic consumption survey standard operating procedures and data collection tools

The Antimicrobial Consumption Survey – Study Details

Antimicrobial Consumption Survey

- We will conduct a survey of antibiotic prescribing in relation to clinical diagnosis in all hospitalized adults, children and neonates across 14 Kenyan hospitals.
- The survey will collate data on drug, dose and indicators of antibiotic (and other antimicrobial) prescribing across inpatients in the participant facilities, with the aim of identifying targets to improving quality of prescribing.
- Relevant data is generally held in clinical patient notes and treatment sheets; the survey team will therefore work with medical record administrators and clinical staff at each ward to collate relevant information.
- The survey will involve reviewing all prescriptions of antibiotics and other antimicrobials among hospitalized individuals, by inspecting treatment sheets, and establishing the diagnosis and relevant investigations that would support the prescription, by inspecting all other relevant clinical patient notes.
- Participant hospitals are committed to facilitate the task of the survey team by providing:
  - Access to - and support from - relevant ward staff,
  - Access to relevant patient notes,
  - Access to a room with tables, chairs and electricity.
- The ACS will follow standard operating procedures and data capture forms currently in use as part of the '*Global Point Prevalence Survey on Antimicrobial Consumption and Resistance (Global PPS)*' and will consequently collate data on additional antimicrobials relevant to Global-PPS activities (i.e. antifungals, antivirals, antimalarials). Data capture forms and details of how the survey will be conducted are given in the following sections. The Global Point Prevalence Survey (PPS) – Year 2017 protocol can be found at http://www.global-pps.com/documents/

Outline Protocol

Key aspects of the protocol are described below:

1. Preparation of a hospital ward list;
2. What guidelines are being used to guide prescription of antibiotics and other antimicrobials based on diagnosis or clinical condition. This information may be available at the facility-level or at the ward-level.
3. Inclusion of all eligible hospital wards in the survey (except for the case of Kenyatta National Hospital, where only neonatal wards will be included in the hospital);
4. Survey of each ward only once on a single day. Different wards may be surveyed on different dates;
5. Inclusion criteria:
   1. All inpatients admitted on the ward before 8 o'clock in the morning on the day of the survey, including all neonate healthy infants in the case of maternity wards (denominator data);
   2. All inpatients *"on antibiotics or other eligible antimicrobials"*, and who were admitted before 8 o'clock in the morning on the day of the survey (numerator data). Patients *"on antibiotics"* are those undergoing antibiotic treatment even if the drug is not administered the day of the survey. The definition also includes patients who received one or more doses of antibiotics or other eligible anti-infective agents intended as surgical prophylaxis in the 24 h prior to 8:00 am on the day of the survey.
6. Exclusion criteria:
   1. All day admissions and outpatients (denominator and numerator). This includes all healthy mothers and babies in a maternity ward, who will be discharged within 24 hours of admission, following successful delivery. These patients should be regarded as day patients even if present in the ward at the time of the survey;
   2. Patients discharged before or admitted after 8 o'clock in the morning on the day of the survey (denominator and numerator);
   3. Inpatients on antibiotics or other eligible antimicrobials, where treatment was no longer active/ongoing at 8 o'clock in the morning on the day of the survey (numerator).
   4. Amenity, Psychiatry, ENT, Renal, ICU, NICU (including in Kenyatta hospital), Neurosurgery and Eye wards are excluded from the survey, as these units are not present in all hospitals.
7. Denominator data:
   1. Total number of admitted eligible patients (i.e. admitted before 8 am) in the ward surveyed;
   2. Total number of available beds attributed to inpatients at 8 am of the ward surveyed (i.e. number of total inpatient beds [occupied plus empty] at the time of the survey).
8. Antibiotics and other eligible antimicrobial agents: Classification of antibiotics and other antimicrobials will agree with the ATC/DDD Index 2017 of the WHO Collaborating Centre for Drug Statistics Methodology;
   1. Inclusion criteria - Anti-infective agents within the following ATC codes:
      1. J01: Antibiotics for systemic use
      2. A07AA: Antibiotics used as intestinal anti-infectives
      3. P01AB: Antiprotozoals used as antibacterial agents, nitroimidazole derivatives
      4. J04A: Antibiotics and other drugs used for treatment of tuberculosis
      5. J05AH: Antivirals used for influenza - Neuraminidase inhibitors
      6. J02 and D01BA: Antimycotics and antifungals for systemic use
      7. P01B: Antimalarials
   2. Exclusion criteria: Antibiotics and other antimicrobials for topical use.
9. Completion of data collection forms:
   1. Ward form (denominator)
   2. Patient form (numerator)
10. To complete 'Patient forms', the investigators will review all [medical, nursing and drug prescription chart] patient records. If the information available is not sufficient surveyor/s may request additional information from nurses, pharmacists or doctors caring for the patient. Searching for information from other sources such a laboratory computer systems, phoning laboratories *etc.,* is not required. At no point shall there be any discussion about the appropriateness (or lack thereof) of the prescribed medication. The ward staff MUST NOT feel evaluated at the individual level.
11. Confidentiality:
    1. Data stored for analysis WILL NOT bear the names of patients or staff and neither patients, staff nor hospitals will be named in any published reports. Inpatient numbers (i.e. Patient IP number) will be removed from the database prior to analysis and following data cleaning. It is not permissible to discuss the information collected with anyone outside the research team.
12. Data entry conventions:
    1. For numerical fields, enter -1 if the information is missing (applicable but not available).
    2. For date fields, enter 01/01/1913 if the information is missing (applicable but not available)
    3. For date fields, enter 01/01/1914 if not applicable
    4. For drop down lists with the option 'empty', choosing 'empty' means that the information requested is not available.
13. Data Entry and Synchronization
    1. After data entry into REDCap, the data clerks will be expected to synchronize the data daily. This is to ensure the data are stored at the KEMRI/Wellcome Trust servers. You will be provided with internet modems to allow synchronization of the data over the internet.

| **Supplement 5: Table of available guidelines by disease** | | | | | |
| --- | --- | --- | --- | --- | --- |
|  | Condition | **Adult/**  **Paediatric** | **Treatment Options** | **Source** | **Reference** |
|  |  |  |  |  |  |
| **1** | Pneumonia | Adult | fluoroquinolone (moxifloxacin, gemifloxacin, or levofloxacin [750 mg]) | Infectious Diseases Society of America; ATS: American Thoracic Society 2019 | Metlay JP, Waterer GW, Long AC, Anzueto A, Brozek J, Crothers K, Cooley LA, Dean NC, Fine MJ, Flanders SA, Griffin MR. Diagnosis and Treatment of Adults with Community-acquired Pneumonia. An Official Clinical Practice Guideline of the American Thoracic Society and Infectious Diseases Society of America. American Journal of Respiratory and Critical Care Medicine. 2019 Oct 1;200(7):e45-67. |
|  |  |  | An antipneumococcal beta-lactam (preferred agents: cefotaxime, ceftriaxone, or ampicillin-sulbactam; or ertapenem for selected patients) PLUS a macrolide (azithromycin, clarithromycin, or erythromycin) |  |  |
|  |  |  | Treat with benzyl penicillin 2 mega units IM IV 6 hourly + gentamicin 240mg IM IV once a day 5 days OR IV ceftriaxone 2g every 24 hours OR erythromycin 500mg 6 hourly for 5 days | Clinical Management and Referral Guidelines – Volume III: Clinical Guidelines for Management and Referral of Common Conditions at Levels 4–6: Hospitals. | Ministry of Medical Services and Ministry of Public Health and Sanitation. Clinical Management and Referral Guidelines – Volume III: Clinical Guidelines for Management and Referral of Common Conditions at Levels 4–6: Hospitals. Nairobi: Ministry of Medical Services and Ministry of Public Health and Sanitation; 2009. |
|  |  |  |  |  |  |
|  |  |  | Amoxicillin 500 mg orally three times daily | 2009 guideline recommendations of the British Thoracic Society | Lim WS, Baudouin SV, George RC, et al. BTS guidelines for the management of community-acquired pneumonia in adults: update 2009. Thorax 2009; 64 Suppl 3:iii1. Copyright © 2009 BMJ Publishing Group Ltd. |
|  |  |  | Amoxicillin 500 mg to 1 gram orally three times daily plus clarithromycin 500 mg orally twice daily |  |  |
|  |  |  | Doxycycline 200 mg loading dose then 100 mg orally or levofloxacin 500 mg orally once daily or moxifloxacin 400 mg orally once daily |  |  |
|  |  |  | amoxicillin 500 mg IV three times daily or benzylpenicillin (penicillin G) 1.2 grams IV four times daily plus clarithromycin 500 mg IV twice daily |  |  |
|  |  | Adult | Amoxicillin/Clavulanic Acid/ cefuroxime or Ceftriaxone and Macrolide | Kenyatta National Hospital Kenya | The KNH Guide to Empiric Antimicrobial Therapy 2018 |
|  |  | Children | benzylpenicillin + gentamicin | Basic Paediatric Protocols for ages up to 5 years. 2016 | Ministry of Health. Basic Paediatric Protocols for ages up to 5 years. 2016 [Available from: http://idoc-africa.org/images/documents/2016/Basic_Paediatric_Protocol_2016/MAY%2023rd%20BPP%202016%20SA.pdf. |
| **2** | Skin and Soft Tissue Infections | Adult/Children | Cloxacillin | Clinical Management and Referral Guidelines – Volume III: Clinical Guidelines for Management and Referral of Common Conditions at Levels 4–6: Hospitals. | Ministry of Medical Services and Ministry of Public Health and Sanitation. Clinical Management and Referral Guidelines – Volume III: Clinical Guidelines for Management and Referral of Common Conditions at Levels 4–6: Hospitals. Nairobi: Ministry of Medical Services and Ministry of Public Health and Sanitation; 2009. |
|  |  | Adult | Amoxicillin/Clavulanic Acid or Clindamycin or Doxycycline | Kenyatta National Hospital Kenya | The KNH Guide to Empiric Antimicrobial Therapy 2018 |
|  |  | Adult (extended hospitalization with invasive procedure ) | Piperacillin/Tazobactam+ Amikacin or Cefipime +Amikacin |  |  |
|  |  |  |  |  |  |
|  |  |  |  |  |  |
|  |  |  |  |  |  |
|  |  |  |  |  |  |
|  |  |  |  |  |  |
|  |  | Adult/Children | Vancomycin +piperacillin | Infectious Diseases Society of America | Dennis L. Stevens, Alan L. Bisno, Henry F. Chambers, E. Patchen Dellinger, Ellie J. C. Goldstein, Sherwood L. Gorbach, Jan V. Hirschmann, Sheldon L. Kaplan, Jose G. Montoya, James C. Wade, Practice Guidelines for the Diagnosis and Management of Skin and Soft Tissue Infections: 2014 Update by the Infectious Diseases Society of America, Clinical Infectious Diseases, Volume 59, Issue 2, 15 July 2014, Pages e10–e52, https://doi.org/10.1093/cid/ciu296 |
|  |  |  | Penicillin, Ceftriaxone, Clindamycin, Cephazolin |  |  |
|  |  |  | Ampicillin - Sulbactam |  |  |
|  |  |  | Ceftriaxone+Metronidazole |  |  |
|  |  |  | Ciprofloxacin+Metronidazole |  |  |
|  |  |  | Levofloxacin+Metronidazole |  |  |
|  |  |  |  |  |  |
|  |  |  |  | National Institute for Health and Care Excellence (NICE) | Guideline on cellulitis and erysipelas – Antimicrobial prescribing (2019)-- https://www.nice.org.uk/guidance/ng141/resources/visual-summary-pdf-6908401837 |
| **3** | Urinary Tract Infections | Adult- Lower UTI | Cotrimoxazole | Clinical Management and Referral Guidelines – Volume III: Clinical Guidelines for Management and Referral of Common Conditions at Levels 4–6 |  |
|  |  | Adult -Upper UTI | Gentamicin, Ciprofloxacin |  | Ministry of Medical Services and Ministry of Public Health and Sanitation. Clinical Management and Referral Guidelines – Volume III: Clinical Guidelines for Management and Referral of Common Conditions at Levels 4–6: Hospitals. Nairobi: Ministry of Medical Services and Ministry of Public Health and Sanitation; 2009. |
|  |  | Adult( Category 1) | Nitrofurantoin or Cefuroxime or Ciprofloxacin | Kenyatta National Hospital Kenya | The KNH Guide to Empiric Antimicrobial Therapy 2018 |
|  |  | Adult (Category 2) | Nitrofurantoin or Etrapenem or Piperacillin/Tazobactam |  |  |
|  |  | Adult (Category 3) | Meropenem or Imipenem+ Amikacin or Piperacillin/Tazobactam+Amikacin |  |  |
|  |  | Adult | Ceftriaxone | Infectious Diseases Society of America/NICE | Thomas M. Hooton, Suzanne F. Bradley, Diana D. Cardenas, Richard Colgan, Suzanne E. Geerlings, James C. Rice, Sanjay Saint, Anthony J. Schaeffer, Paul A. Tambayh, Peter Tenke, Lindsay E. Nicolle, Diagnosis, Prevention, and Treatment of Catheter-Associated Urinary Tract Infection in Adults: 2009 International Clinical Practice Guidelines from the Infectious Diseases Society of America, Clinical Infectious Diseases, Volume 50, Issue 5, 1 March 2010, Pages 625–663, https://doi.org/10.1086/650482 |
|  |  |  | Ciprofloxacin |  |  |
|  |  |  | Levofloxacin |  |  |
|  |  |  | Trimethoprim-Sulfamethoxazole |  |  |
|  |  |  | Amoxicillin Clavulanate |  |  |
|  |  | Children | Co-amoxiclav Cefuroxime Ceftriaxone Gentamicin Amikacin | National Institute for Health and Care Excellence (NICE) | Pyelonephritis (acute): antimicrobial prescribing https://www.nice.org.uk/guidance/ng111/resources/visual-summary-pdf-6544161037 UTI (catheter): antimicrobial prescribing |
|  |  | Adult | Ceftriaxone, Ciprofloxacin,  Levofloxacin, | National Institute for Health and Care Excellence (NICE) | UTI (catheter): antimicrobial prescribing https://www.nice.org.uk/guidance/ng113/resources/visual-summary-pdf-6599495053 |
|  |  | Lower UTI | Nitrofurantoin | National Institute for Health and Care Excellence (NICE) | UTI (lower): antimicrobial prescribing https://www.nice.org.uk/guidance/ng109/resources/visual-summary-pdf-6544021069 |
| **4** | Intrabdominal Infections | Adults(Category 1) | Ceftriaxone+Metronidazole or Ciprofloxacin+Metronidazole or Tigecycline+ Metronidazole | Kenyatta National Hospital Kenya | The KNH Guide to Empiric Antimicrobial Therapy 2018 |
|  |  | Adults(Category 2) | Etrpenem or Piperacillin/Tazobactam+  Aminoglycoside or Ceftazidime+Aminoglycoside+  Metronidazole or Tigecycline+ Metronidazole |  |  |
|  |  | Adults(Category 2) | Imipenem+/-Aminoglycoside or Meropenem +/- aminoglycoside or cefipime+ aminoglycoside+metronidazole |  |  |
|  |  | Children | Ceftriaxone, cefotaxime, cefepime, or ceftazidime, each in combination with metronidazole; gentamicin or tobramycin, each in combination with metronidazole or clindamycin, and with or without ampicillin | Infectious Diseases Society of America | Joseph S. Solomkin, John E. Mazuski, John S. Bradley, Keith A Rodvold, Ellie J.C. Goldstein, Ellen J. Baron, Patrick J. O'Neill, Anthony W. Chow, E. Patchen Dellinger, Soumitra R. Eachempati, Sherwood Gorbach, Mary Hilfiker, Addison K. May, Avery B. Nathens, Robert G. Sawyer, John G. Bartlett, Diagnosis and Management of Complicated Intra-abdominal Infection in Adults and Children: Guidelines by the Surgical Infection Society and the Infectious Diseases Society of America, Clinical Infectious Diseases, Volume 50, Issue 2, 15 January 2010, Pages 133–164, https://doi.org/10.1086/649554 |
|  |  | Adults | Cefazolin, cefuroxime, ceftriaxone, cefotaxime, ciprofloxacin, or levofloxacin, each in combination with metronidazole |  |  |
| **5** | CNS Infections | Adults | Benzylpenicillin 4 mega units IV 6 hourly for 14– 21 days OR chloramphenicol 1g IV 6 hourly for 14 days, OR ceftriaxone 24g/ day IV 12 hourly for 14–21 days, Vancomycin 2g/day IV 8–12 hourly OR meropenem 2g/day IV 8 hourly | Clinical Management and Referral Guidelines – Volume III: Clinical Guidelines for Management and Referral of Common Conditions at Levels 4–6: | Ministry of Medical Services and Ministry of Public Health and Sanitation. Clinical Management and Referral Guidelines – Volume III: Clinical Guidelines for Management and Referral of Common Conditions at Levels 4–6: Hospitals. Nairobi: Ministry of Medical Services and Ministry of Public Health and Sanitation,; 2009. |
|  |  | Children | Ceftriaxone | Basic Paediatric Protocols for ages up to 5 years. 2016 | Ministry of Health. Basic Paediatric Protocols for ages up to 5 years. 2016 [Available from: http://idoc-africa.org/images/documents/2016/Basic_Paediatric_Protocol_2016/MAY%2023rd%20BPP%202016%20SA.pdf. |
|  |  | Adults | Vancomycin plus ampicillin plus a third-generation cephalosporin | Infectious Disease Society of America | *Liu C, Bayer A, Cosgrove SE, et al. Clinical Practice Guidelines by the Infectious Diseases Society of America for the Treatment of Methicillin-Resistant Staphylococcus Aureus Infections in Adults and Children: Executive Summary. Clin Infect Dis 2011; 52:285.* |
|  |  | Adults >50 | Vancomycin plus ampicillin plus a third-generation cephalosporin |  |  |
|  |  | Adults Post Trauma | Vancomycin plus cefepime; OR vancomycin plus ceftazidime; OR vancomycin plus meropenem |  |  |
| **6** | Sepsis/Septic Shock | Adults | vancomycin with one of the following:  3rd generation (e.g., ceftriaxone or cefotaxime) or 4th generation cephalosporin (cefepime), or   beta-lactam/beta-lactamase inhibitor (eg, piperacillin-tazobactam, ticarcillin-clavulanate), or   carbapenem (e.g., imipenem or meropenem) | Surviving Sepsis Campaign | Rhodes A, Evans LE, Alhazzani W, Levy MM, Antonelli M, Ferrer R, et al. Surviving Sepsis Campaign: International Guidelines for Management of Sepsis and Septic Shock: 2016. Intensive Care Medicine. 2017;43(3):304-77. |
|  |  | Children | Vancomycin (15 mg/kg, maximum 1 to 2 g, for the initial dose) PLUS cefotaxime (100 mg/kg, maximum 2 g, for the initial dose) OR ceftriaxone (75 mg/kg, maximum 2 g, for the initial dose)  •Consider adding an aminoglycoside (e.g., gentamicin) for possible GU source and/or piperacillin with tazobactam, clindamycin or metronidazole for possible GI source | Surviving Sepsis Campaign | Rhodes A, Evans LE, Alhazzani W, et al. Surviving Sepsis Campaign: International Guidelines for Management of Sepsis and Septic Shock: 2016. Intensive Care Med 2017; 43:304. |
| **7** | Bone and Joint Infection | Adults/Children | Clindamycin( Adults),Cloxacillin + chloramphenicol Cloxacillin + gentamicin or Amoxicillin + gentamicin for septic arthritis | Clinical Management and Referral Guidelines – Volume III: Clinical Guidelines for Management and Referral of Common Conditions at Levels 4–6: | Ministry of Medical Services and Ministry of Public Health and Sanitation. Clinical Management and Referral Guidelines – Volume III: Clinical Guidelines for Management and Referral of Common Conditions at Levels 4–6: Hospitals. Nairobi: Ministry of Medical Services and Ministry of Public Health and Sanitation; 2009. |
|  |  | Adults | Vancomycin PLUS a third- or fourth-generation cephalosporin (such as ceftriaxone, ceftazidime, or cefepime) | Infectious Disease Society of America | Mader JT, Cantrell JS, Calhoun J. Oral ciprofloxacin compared with standard parenteral antibiotic therapy for chronic osteomyelitis in adults. J Bone Joint Surg Am 1990; 72:104.Davey PG, Rowley DR, Phillips GA. Teicoplanin--home therapy for prosthetic joint infections. Eur J Surg Suppl 1992; :23.Lazzarini L, Lipsky BA, Mader JT. Antibiotic treatment of osteomyelitis: what have we learned from 30 years of clinical trials? Int J Infect Dis 2005; 9:127.Black J, Hunt TL, Godley PJ, Matthew E. Oral antimicrobial therapy for adults with osteomyelitis or septic arthritis. J Infect Dis 1987; 155:968.Gentry LO, Rodriguez-Gomez G. Ofloxacin versus parenteral therapy for chronic osteomyelitis. Antimicrob Agents Chemother 1991; 35:538.Lew DP, Waldvogel FA. Quinolones and osteomyelitis: state-of-the-art. Drugs 1995; 49 Suppl 2:100. |
|  |  | Children | Vancomycin PLUS Nafcillin or Ofloxacin PLUS a third- or fourth-generation cephalosporin (such as ceftriaxone, ceftazidime, or cefepime) and Clindamycin |  |  |
| **8** | Post Caesarean Section Prophylaxis | Adults | First Generation Cephalosporin or Add metronidazole, azithromycin, or doxycycline to a first- or second-generation cephalosporin | Infectious Disease Society of America | Dale W. Bratzler, E. Patchen Dellinger, Keith M. Olsen, Trish M. Perl, Paul G. Auwaerter, Maureen K. Bolon, Douglas N. Fish, Lena M. Napolitano, Robert G. Sawyer, Douglas Slain, James P. Steinberg, Robert A. Weinstein, Clinical practice guidelines for antimicrobial prophylaxis in surgery, American Journal of Health-System Pharmacy, Volume 70, Issue 3, 1 February 2013, Pages 195–283, https://doi.org/10.2146/ajhp120568 |
| **9** | Prophylaxis for bone and joint surgery | Adults | Cephazolin, Clindamycin, vancomycin | Infectious Disease Society of America | Dale W. Bratzler, E. Patchen Dellinger, Keith M. Olsen, Trish M. Perl, Paul G. Auwaerter, Maureen K. Bolon, Douglas N. Fish, Lena M. Napolitano, Robert G. Sawyer, Douglas Slain, James P. Steinberg, Robert A. Weinstein, Clinical practice guidelines for antimicrobial prophylaxis in surgery, American Journal of Health-System Pharmacy, Volume 70, Issue 3, 1 February 2013, Pages 195–283, https://doi.org/10.2146/ajhp120568 |
| **10** | Prophylaxis for GIT surgery | Adults | Cefazolin, cefoxitin, cefotetan, ceftriaxone,ampicillin–sulbactam, Clindamycin or vancomycin +aminoglycoside or aztreonam orfluoroquinolone or Metronidazole + aminoglycoside or fluoroquinolone | Infectious Disease Society of America | Dale W. Bratzler, E. Patchen Dellinger, Keith M. Olsen, Trish M. Perl, Paul G. Auwaerter, Maureen K. Bolon, Douglas N. Fish, Lena M. Napolitano, Robert G. Sawyer, Douglas Slain, James P. Steinberg, Robert A. Weinstein, Clinical practice guidelines for antimicrobial prophylaxis in surgery, American Journal of Health-System Pharmacy, Volume 70, Issue 3, 1 February 2013, Pages 195–283, https://doi.org/10.2146/ajhp120568 |
| **11** | Prophylaxis for Urological Surgery | Adults | Cefazolin ± aminoglycoside, cefazolin ± aztreonam, ampicillin–sulbactam, Fluoroquinolone, trimethoprim–sulfamethoxazole, Cefazolin + metronidazole, cefoxitin, aminoglycoside + metronidazole or clindamycin | Infectious Disease Society of America | Dale W. Bratzler, E. Patchen Dellinger, Keith M. Olsen, Trish M. Perl, Paul G. Auwaerter, Maureen K. Bolon, Douglas N. Fish, Lena M. Napolitano, Robert G. Sawyer, Douglas Slain, James P. Steinberg, Robert A. Weinstein, Clinical practice guidelines for antimicrobial prophylaxis in surgery, American Journal of Health-System Pharmacy, Volume 70, Issue 3, 1 February 2013, Pages 195–283, https://doi.org/10.2146/ajhp120568 |
| **12** | Neonatal Infections | Neonates | Penicillin+ Gentamicin, Ceftazidime, Metronidazole, Ceftriaxone, Cefotaxime | Basic Paediatric Protocols for ages up to 5 years. 2016 | Ministry of Health. Basic Paediatric Protocols for ages up to 5 years. 2016 [Available from: http://idoc-africa.org/images/documents/2016/Basic_Paediatric_Protocol_2016/MAY%2023rd%20BPP%202016%20SA.pdf. |
| **13** | Genitourinary system |  |  | Ministry of Health Kenya | Ministry of Health. Kenya National Guidelines for Prevention, Management and Control of Sexually Transmitted Infections. Nairobi: National AIDS and STI Control Programme; 2018. |
|  |  | Male STD | IM ceftriaxone/Gentamicin and Azithromycin |  |  |
|  |  | Cervicitis | IM ceftriaxone/Gentamicin and Azithromycin |  |  |
|  |  | Pelvic Inflammatory Disease | Cefixime/Ceftriaxone/Gentamicin and Doxycline and Metronidazole |  |  |

Supplement 6: Heat map showing the physical availability of guidelines by disease condition across the departments


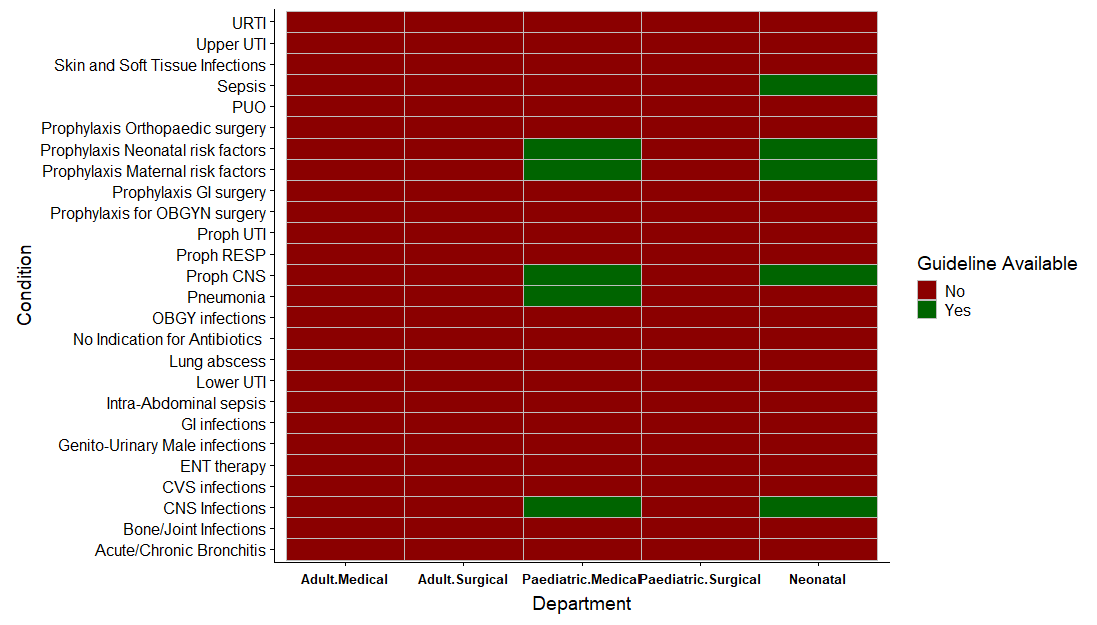

Supplement: Supplementary file 1 [file mmc1.docx]
